# Position Statement on Active Outdoor Play

**DOI:** 10.3390/ijerph120606475

**Published:** 2015-06-08

**Authors:** Mark S. Tremblay, Casey Gray, Shawna Babcock, Joel Barnes, Christa Costas Bradstreet, Dawn Carr, Guylaine Chabot, Louise Choquette, David Chorney, Cam Collyer, Susan Herrington, Katherine Janson, Ian Janssen, Richard Larouche, William Pickett, Marlene Power, Ellen Beate Hansen Sandseter, Brenda Simon, Mariana Brussoni

**Affiliations:** 1 Healthy Active Living and Obesity Research Group, Children’s Hospital of Eastern Ontario Research Institute, 401 Smyth Road, Ottawa, Ontario, K1H 8L1, Canada; E-Mails: casgray@cheo,on.ca (C.G.); jbarnes@cheo.on.ca (J.B.); rlarouche@cheo.on.ca (R.L.); 2 Department of Pediatrics, Children’s Hospital of Eastern Ontario, University of Ottawa, 401 Smyth Road, Ottawa, Ontario, K1H 8L1, Canada; 3 School of Human Kinetics, University of Ottawa, 125 University Private, Ottawa, Ontario, K1N 1A2, Canada; 4 KidActive, 559A, Burtron Lane, Combermere, Ontario, K0J 1L0, Canada; E-Mail: sbabcock@kidactive.ca; 5 ParticipACTION, 77 Bloor Street West, Toronto, Ontario, M5S 1M2, Canada; E-Mails: ccbradstreet@participACTION.com (C.C.B.); kjanson@participACTION.com (K.J.); 6 Canadian Parks Council, 455 Hunter Street West, Peterborough, Ontario, K9H 2M7, Canada; E-Mail: dcarr@parks-parcs.ca; 7 Evaluation Platform on Obesity Prevention, Quebec Heart and Lung Research Institute, Laval University, 2725 Chemin Ste-Foy, Québec City, Québec, G1V 4G5, Canada; E-Mail: guylaine.chabot@criucpq.ulaval.ca; 8 Best Start Resource Centre—Health Nexus, 180 Dundas Street West, Toronto, Ontario, M5G 1Z8, Canada; E-Mail: l.choquette@healthnexus.ca; 9 PHE Canada, 301-2197 Riverside Drive, Ottawa, Ontario, K1H 7X3, Canada; E-Mail: dchorney@ualberta.ca; 10 Evergreen, 550 Bayview Avenue, Toronto, Ontario, M4W 3X8, Canada; E-Mail: ccollyer@evergreen.ca; 11 School of Architecture and Landscape Architecture, University of British Columbia, 379-2357 Main Mall, Vancouver, British Columbia, B6T 1Z4, Canada; E-Mail: susan.herrington@ubc.ca; 12 Department of Public Health Sciences, School of Kinesiology and Health Studies, Queen’s University, 99 University Avenue, Kingston, Ontario, K7L 2P5, Canada; E-Mail: ian.janssen@queensu.ca; 13 Department of Public Health Sciences, Carruthers Hall, Queen’s University, Kingston, Ontario, K7L 2P5, Canada; E-Mail: will.pickett@queensu.ca; 14 Forest School Canada, Child and Nature Alliance of Canada, 411 Corkstown Road, Ottawa, Ontario, K2K 2Y1, Canada; E-Mail: mpower@forestschoolcanada.ca; 15 Department of Physical Education and Health, College of Early Childhood Education, Queen Maud University, Thrond Nergaardsvei 7, NO-7044 Trondheim, Norway; E-Mail: ebs@dmmh.no; 16 PLAYbynature, 226 Albany Avenue, Toronto, Ontario, M5R 3C6, Canada; E-Mail: brenda@playbynature.org; 17 British Columbia Injury Research & Prevention Unit, Department of Pediatrics, School of Population & Public Health, Child & Family Research Institute, British Columbia Children’s Hospital, University of British Columbia, , F511-4480 Oak Street, Vancouver, British Columbia, V6H 3V4, Canada; E-Mail: mbrussoni@cw.bc.ca

**Keywords:** physical activity, health promotion, risk taking, safety, injury, playground

## Abstract

A diverse, cross-sectorial group of partners, stakeholders and researchers, collaborated to develop an evidence-informed *Position Statement* on active outdoor play for children aged 3–12 years. The *Position Statement* was created in response to practitioner, academic, legal, insurance and public debate, dialogue and disagreement on the relative benefits and harms of active (including risky) outdoor play. The *Position Statement* development process was informed by two systematic reviews, a critical appraisal of the current literature and existing position statements, engagement of research experts (N = 9) and cross-sectorial individuals/organizations (N = 17), and an extensive stakeholder consultation process (N = 1908). More than 95% of the stakeholders consulted strongly agreed or somewhat agreed with the *Position Statement*; 14/17 participating individuals/organizations endorsed it; and over 1000 additional individuals and organizations requested their name be listed as a supporter. The final *Position Statement on Active Outdoor Play* states: “Access to active play in nature and outdoors—with its risks— is essential for healthy child development. We recommend increasing children’s opportunities for self-directed play outdoors in all settings—at home, at school, in child care, the community and nature.” The full *Position Statement* provides context for the statement, evidence supporting it, and a series of recommendations to increase active outdoor play opportunities to promote healthy child development.

## 1. Introduction

There is global concern over the progressive trend towards lifestyles that are conducive to the promotion of non-communicable diseases [1,2]. Childhood obesity and inactivity are prominently featured in this challenge, requiring solutions that take a life-course approach, emphasizing the importance of cumulative physical, social and cultural exposures [1,2,3,4,5]. The prevalence of childhood obesity worldwide is at an all-time high and increasing [6]. The proportion of children around the world meeting physical activity guidelines is very low and likely declining [7,8]. This is troubling as physical activity and sedentary behaviour habits formed during childhood tend to track over time into adulthood [9,10]. The decreased opportunity for, and prioritization of, childhood healthy active living and active outdoor play has been positioned as a child rights concern [11]. The issue is of sufficient urgency that the Director General of the World Health Organization recently established a *Commission on Ending Childhood Obesity* to provide recommendations for preventing and managing childhood obesity in a variety of contexts around the world [12,13]. Finding strategies to promote healthy child development through the prevention of obesity and inactivity are required. Approaches that are accessible, acceptable, culturally adaptable, feasible, cost-effective, and scalable are a priority worldwide. 

Healthy child development is influenced by many individual, family, school, community, political and global factors that interact over time [14]. Several substantive societal changes have occurred across these levels of influence in recent years, including where and how children spend their discretionary time. For example, there is empirical, observational and intuitive evidence that children today play outside less than their parents did and that children’s play has become more structured and occurs increasingly indoors [15,16,17,18,19,20,21,22]. Reported levels of screen time in developed countries suggest that sedentary indoor time is high amongst most children and youth today [17,18,19,20,21,23,24]. Canadian children now spend an average of 8.6 hours per day sedentary [25]. The lure of the indoors has been fueled by improvements in indoor climate control and increased entertainment temptations (e.g., television, computers, electronic games, tablets, cell phones) [26], as well as concerns for child safety [16,17,27,28]. This drift from time spent outdoors to increased time spent indoors has occurred concurrent with the childhood obesity and inactivity crisis, and parental expectations surrounding child protection. 

Academic debate and public discussion about the relative benefits and harms of active outdoor play is captured in ubiquitous stories, blogs, and articles about climbing trees, tobogganing safety, sun-safety, playground safety, appropriate age and level of supervision of free-range children, and social norms around acceptable distances children can wander/roam unsupervised. Some argue that we have become an excessively risk averse society, to the extent that we have limited essential learning and developmental opportunities for children, while also reducing their physical activity and increasing sedentary behaviours. Others believe that safety guidelines and standards, child protection services and contemporary use of the precautionary principle have improved healthy child development through injury prevention and a reduction in exposure to harm. This debate is active in the published literature [29,30,31,32]. The 2012 Active Healthy Kids Canada Report Card extolled the benefits of self-directed active outdoor play [18] while the Canadian Paediatric Society Position Statement on preventing playground injuries recommends active supervision [33]. The debate is often polarized and supporting evidence selectively cited. Fears of legal and/or insurance consequences often motivate decision-makers to default to elimination of risks or what has been called surplus safety [34]. However, recent efforts in Canada [35] and England [36] have promoted a more balanced approach to risk and safety in childhood by bringing divergent professions in the area of health promotion together through reasoned argument and debate, as opposed to polarizing confrontations. Ungar noted that parents are not swayed by statistics indicating that their communities are safe and that there is a low likelihood of adverse outcomes from being outdoors [37]. Rather, most parents are motivated to reduce their control and supervision and increase their willingness to allow children’s age-appropriate risk-taking and responsibility when they are persuaded that their actions place their child at a disadvantage. 

The underlying premise that the indoors is safer than the outdoors however may be misguided in light of the potential harms of the internet (e.g., violence, cyber-bullying, on-line predators, pornography), unnecessary incidental eating prompted by advertisements or convenience, reduced physical activity and even the hazards of indoor air [38,39,40,41,42,43]. Regardless, there is a need for a balanced review of the evidence and a coming together of relevant stakeholders to agree upon an evidence-informed foundation from which responsible initiatives can emanate to promote healthy child development. To address this need a group of researchers and Canadian organizations (paper authors) agreed to work together to create a *Position Statement on Active Outdoor Play* informed by the research literature, expert consensus and practitioner experience. It is recognized that lack of a common understanding and use of key terms can complicate this debate. For example, risk is often interpreted as a bad thing, yet exposure to risk has been shown to be essential for healthy child development [32,44,45,46,47,48]. Consequently, operational definitions of key terms used in, and related to, the *Position Statement* are provided in Table 1.

**Table 1:** Operational definitions of key terms.

Term	Operational Definition
Active Outdoor Play	Active outdoor play, sometimes referred to as active free-play or self-directed play is defined here as, “unstructured physical activity that takes place outdoors in the child’s free time [16].”
Harm	Physical or mental damage or injury: something that causes someone or something to be hurt, broken, made less valuable or successful [49]
Hazard/ Danger	A source of harm that is not obvious to the child, such that the potential for injury is hidden [32,47]. The potential for injury can be immediate or long term. A source of danger [50]
Hyper-parenting	“‘Hyper-parenting,’ ‘invasive parenting,’ or ‘intensive parenting,’ in which a climate of ‘inflated risk’ leads parents to micromanage all aspects of their children’s lives in an effort to protect the child from adverse experiences” [48]. “Parents attempt to become experts on optimal parenting strategies, and child health and development so as to ensure that their children achieve their full potential” [51]. “A variety of different types of highly involved parents (from ‘‘helicopter parents’’ to ‘‘tiger moms’’)” [52]
Joint and Several Liability Reform	Joint and Several Liability is a legal principle that permits the injured party in a tort action to recover the entire amount of compensation due for injuries from any tortfeasor who is able to pay, regardless of the degree of that party’s negligence [53]. Entities that are often viewed as those with the greatest amount of liability insurance are seeking reform to this principle so that the amount they pay towards an injured party directly correlates with the degree to which they were negligent.
Natural Environments	Environments that include natural elements such as plants, soil, and water. These may be human made (e.g., gardens, nature playgrounds and urban parks) or wild and naturally occurring (e.g., wooded areas, meadows and beaches).
Nature	“The phenomena of the physical world collectively, including plants, animals, the landscape, and other features and products of the earth, as opposed to humans or human creations” [54].
Physical Activity	“Any body movement produced by skeletal muscles resulting in a substantial increase over resting energy expenditure” [55].
Public Entities	Municipal governments, regional governments, local economic development legal entities or authorities, sectorial representative organizations.
Risky Play	Thrilling and exciting play that can include the possibility of physical injury. Types of risky play include play at height, speed, near dangerous elements (e.g., water, fire), with dangerous tools, rough and tumble play (e.g., play fighting), and where there is the potential for disappearing or getting lost [44,56,57].
Sedentary Behaviour	“Any waking activity characterized by an energy expenditure ≤1.5 metabolic equivalents and a sitting or reclining posture” [58].

This initiative represents a robust, comprehensive, transparent, multi-disciplinary effort to examine the evidence linking active outdoor play, including risky play, to the promotion of healthy active living among Canadian children. The purpose of this paper is to describe the process used to develop the *Position Statement on Active Outdoor Play* and present, explain, substantiate and discuss the final *Position Statement*. 

## 2. Methods

The development and release of the *Position Statement* included securing funding, the completion of two systematic literature reviews, the establishment of a multi-disciplinary consensus group, an extensive stakeholder survey and consultation process, an opportunity for supportive stakeholders to be acknowledged, a comprehensive media, public relations and communication plan, a coordinated dissemination strategy, and proactive planning for follow-up action.

This project was initiated by the Healthy Active Living and Obesity Research Group (HALO; www.haloresearch.ca) at the Children’s Hospital of Eastern Ontario (CHEO) Research Institute. Funding for the systematic reviews was secured through a research grant from The Lawson Foundation and an internal prioritization of funds within HALO. Funding and in-kind support for the development of the *Position Statement* was provided by The Lawson Foundation, HALO, the KidActive—Healthy Kids, Communities and Outdoors grant from the Ontario Ministry of Health and Long-Term Care Healthy Communities Fund (HCF) Program Stream, and ParticipACTION. All participating individuals and organizations also provided in-kind support for the development, dissemination and promotion of the *Position Statement*.

Two systematic reviews were undertaken to help inform the *Position Statement*. Both systematic reviews were registered with the international prospective register of systematic reviews PROSPERO network (registration nos. CRD42014009307 and CRD42014006838) and followed the PRISMA statement for reporting systematic reviews [59]. The reviews investigated the evidence on: (1) the relationship between outdoor time and physical activity, sedentary behaviour, and physical fitness in children [43] and (2) the relationship between risky outdoor play and health in children [46]. 

A small Steering Committee with representatives from each of the project funders was formed to identify individuals and organizations to form a multi-disciplinary consensus group. The final participants in the consensus group and their home organizations are presented Table 2. The consensus group met for 2 days in January, 2015, at Evergreen Brick Works in Toronto where results of the systematic reviews were presented, other research evidence was discussed, the content for the *Position Statement* was determined, plans for a stakeholder consultation survey were initiated, and initial communications and dissemination strategies were formulated. Immediately following the consensus group meeting Katherine Janson, Casey Gray and Mark Tremblay created an initial draft of the *Position Statement* that was circulated to the group. After multiple iterations and revisions, consensus (defined *a priori* as 75% agreement) was achieved on a draft *Position Statement* for the stakeholder consultation. The draft *Position Statement* was translated into French and the translation was verified by Guylaine Chabot, Louise Choquette and Richard Larouche.

**Table 2:** *Position Statement* development consensus group.

Consensus Group Participant	Sector	Home Organization
Mark Tremblay (Chair)	Physical activity research	HALO (www.haloresearch.ca)
Casey Gray (Project Manager)	Physical activity research	HALO (www.haloresearch.ca)
Shawna Babcock	Healthy children and communities	KidActive (www.kidactive.ca)
Mariana Brussoni	Risk and safety research	University of British Columbia (spph.ubc.ca/person/mariana-brussoni/)
Dawn Carr	Parks	Canadian Parks Council (www.parks-parcs.ca/)
Guylaine Chabot	Community health research	Laval University (iucpq.qc.ca/fr/recherche)
Louise Choquette	Early childhood development	Health Nexus (www.healthnexus.ca)
David Chorney	Outdoor Education	PHE Canada (www.phecanada.ca)
Cam Collyer	Green cities	Evergreen (www.evergreen.ca)
Christa Costas Bradstreet	Public health	ParticipACTION (www.participaction.com)
Shannon Devane *	Municipal insurance	OMEX (www.omex.org)
Pamela Fuselli *	Injury prevention	Parachute (www.parachutecanada.org)
Susan Herrington	Landscape architecture research	University of British Columbia (www.sala.ubc.ca/people/faculty/susan-herrington)
Katherine Janson	Health communications	ParticipACTION (www.participaction.com)
Ian Janssen	Physical activity research	Queen’s University (www.queensu.ca/skhs/faculty-and-staff/faculty/ian-janssen)
Richard Larouche	Active transportation research	HALO (www.haloresearch.ca)
Claire LeBlanc *	Pediatrician	Canadian Paediatric Society (www.cps.ca)
Will Pickett	Injury prevention research	Queen’s University (www.queensu.ca/phs/will-pickett)
Marlene Power	Forest schools/outdoor education	Child and Nature Alliance of Canada and Forest Schools Canada (www.childnature.ca)
Ellen Sandseter	Risky play research	Queen Maud University College (Norway) (dmmh.no/en)
Brenda Simon	Lawyer and nature play advocate	PLAYbynature (www.playbynature.org)
Christine Alden (Observer) **	Philanthropy	The Lawson Foundation (www.lawson.ca)

* Shannon Devane (OMEX), Pamela Fuselli (Parachute Canada) and Claire LeBlanc (Canadian Pediatric Society) are not co-authors as directed by their respective organizations (see Results for more details); ** Christine Alden represented The Lawson Foundation who was a significant funder of this project, but she was not a participating member of the consensus group.

An on-line survey was developed to solicit assessments and comments from stakeholders on the draft *Position Statement*. The Children’s Hospital of Eastern Ontario Research Ethics Board reviewed and approved the administration of the survey and use of a passive consent process. The survey sought assessments of the clarity of the various sections of the *Position Statement* as well as levels of agreement with the text. Basic demographic information was requested and an opportunity was provided to offer open comments on any aspect of the *Position Statement*. The complete survey in both English and French is available in Supplemental 1. The survey was disseminated through the various networks of consensus group members, and followed a snowball sampling methodology (recognizing the inherent risk of bias) to maximize reach and input. The survey was live from 25 February to 15 March 2015. After the survey closed, empirical responses were tabulated and analyzed by Joel Barnes. Written comments were consolidated into themes by Casey Gray with assistance from additional HALO staff. A subcommittee of the consensus group, including Mark Tremblay, Casey Gray, Mariana Brussoni, Susan Herrington, Guylaine Chabot, Katherine Janson and Richard Larouche reviewed the survey materials and worked together to revise the *Position Statement* based on the survey feedback, provided changes remained true to the available evidence-base (see Results). The revised *Position Statement* was circulated to the entire consensus group for comment and final revisions. Consensus was achieved on the final *Position Statement*. Revisions were translated to finalize the French version.

The stakeholder survey permitted respondents to express their interest in publicly disclosing their support for the *Position Statement* pending their review of the final draft. To facilitate this, interested respondents were asked to provide an email address where the final *Position Statement* could be sent. Respondents then had the opportunity to review and respond with their permission to publicly display their support/endorsement of the *Position Statement*. The list of supporters is housed on the HALO website (www.haloresearch.ca/OutdoorPlay). 

To maximize the impact of the *Position Statement* a comprehensive media, public relations and communication plan was developed and led by ParticipACTION. A public launch date of 9 June 2015 was established, including a national press release with proactive media outreach in key markets across Canada. To enhance the national outreach, a network of national spokespersons was trained to field interview requests across Canada, including photo opportunities and on-site interviews at adventure playgrounds or similar sites in major markets. Organizations involved in the development of the *Position Statement* received spokesperson training on the key messages and employed template press releases to customize and use to respond to the national release in a coordinated fashion. 

As a part of the communications plan, a comprehensive, coordinated dissemination strategy was put in place by ParticipACTION, utilizing and maximizing the reach of the consensus group, their networks and memberships. The Active Canada 20/20 (www.activecanada2020.ca) Active Play Action Group embraced the Position Statement as its primary activity for the past year to further support its distribution and uptake. The dissemination of the *Position Statement* was significantly enhanced by inserting it in the ParticipACTION Report Card on the Physical Activity of Children and Youth (www.ParticipACTION.com). Formerly the Active Healthy Kids Canada Report Card (www.activehealthykids.ca), the Report Card has enjoyed extraordinary media coverage and impact over the past decade [60]. The strategy to combine the launch of the *Position Statement* and the release of the 2015 Report Card was intended to amplify the reach of each product while also being mutually supportive in message.

The *Position Statement* is a concise, public-facing, consolidation of evidence, and while it does provide recommendations, it is not a program to increase active outdoor play for children. It was designed to be a foundation of credible evidence from which programs, strategies, campaigns, policies, practices and environmental modifications can be provoked, initiated and supported. Nevertheless, deliberate, proactive strategic planning by consensus panel members occurred throughout the *Position Statement* development process such that “responses” to the *Position Statement* in the form of mechanisms to facilitate progress towards the recommendations can be made. The legacy of this initiative is preserved in the *Position Statement* itself as well as this mini-series of papers in the *International Journal of Environmental Research and Public Health* which includes the two background systematic reviews [43,46] and this process and outcomes paper.

## 3. Results

The process, while intensive, was successful and consensus was achieved on the final product. All individuals/organizations represented in the consensus group approved and supported the final *Position Statement* except for Parachute Canada (www.parachutecanada.org/), the Canadian Paediatric Society (www.cps.ca) and OMEX (www.omex.org). Each organization sent a statement to explain the reason they chose not to endorse the final statement. These statements are provided as received below.

- “Parachute very much appreciated the process and are supportive of the work of this group. After a review by our Expert Advisory Committee, unfortunately Parachute is not able to endorse the position statement as it is currently written. One particular area of concern was the way the reference to the CSA standards for playgrounds was worded”.
- “The Canadian Paediatric Society enthusiastically supports the promotion of outdoor play. However, the CPS is concerned that the statement as written does not strike an appropriate balance between encouraging children’s self-directed outdoor activity and appropriate risk reduction. We also note that the broad scope of the recommendations may hamper their implement ability. For this reason, we are unable to endorse it”.
- “OMEX was pleased to participate in the process and supports the spirit and intent of the Position Statement. We agree with Parachute's position with respect to the wording regarding CSA standards. Our role as insurers and risk managers is to promote safety and prevention of risk which conflicts with what the paper is promoting for children play in public places *i.e*., reducing guidelines and standards”.

The final *Position Statement on Active Outdoor Play* is presented in Supplementary 2. The *Position Statement* is supported by peer-reviewed research evidence as cited in Supplementary 2, with complete references also provided in this manuscript. Below, each evidence statement from the *Position Statement* is elaborated on, substantiated, and more details of the supporting evidence are provided. 

### 3.1. Position Statement Evidence

“When children are outside they move more, sit less and play longer—behaviours associated with improved cholesterol levels, blood pressure, body composition, bone density, cardiorespiratory and musculoskeletal fitness and aspects of mental, social and environmental health.”

There is consistent evidence showing that children are more active when they are outside compared to when they are indoors [43,61,62,63,64,65,66,67,68]. Furthermore, when children are outside, they spend less time sedentary compared to when they are indoors [43,64,66,67]. To our knowledge, no study has found outdoor time to be associated with either lower physical activity or increased sedentary time.

There is consistent evidence indicating that physical activity among children and youth is associated with health benefits such as a more favorable lipid profile, lower blood pressure, lower body mass index and waist circumference, and improved cardiorespiratory and musculoskeletal fitness [69,70,71,72,73]. In addition, prospective studies have shown that physical activity (especially weight-bearing activities such as running, jumping and gymnastics) fosters the development of bone mineral density among children and youth [74,75]. Conversely, a systematic review of 232 studies concluded that children and youth who watched TV for more than 2 hours/day had less favorable body composition, reduced fitness, lower scores for self-esteem and pro-social behavior, and decreased academic achievement [76]. Finally, reducing motorized travel can decrease greenhouse gas emissions [77], which has been shown to prevent acute asthma events among children [78].

“Outdoor play is safer than you think! The odds of total stranger abduction are about 1 in 14 million based on RCMP reports. Being with friends outdoors may further reduce this number.”

Concerns about child abduction are frequently cited as a major deterrent by parents to letting their children play and roam outside without supervision [27,79,80]. Furthermore, 45% of parents in one study expressed the belief that abduction is the greatest danger faced by school aged children [81]. ‘Stranger danger’ is not a concern reserved for young children—81% of parents of 10–12 year olds expressed worry about ‘stranger danger’ in another study [79]. Thankfully, these perceptions are not supported by the data. Dalley and Ruscoe [82] reported that in a period of two years across Canada, only 1 case of child abduction by a total stranger was confirmed by the Royal Canadian Mounted Police.

“Broken bones and head injuries unfortunately do happen, but major trauma is uncommon. Most injuries associated with outdoor play are minor.”

Head entrapment and strangulation were once the main causes of death on playgrounds. As a result of efforts by the injury prevention community to reduce hazards where children play, these are now extremely rare [33,83]. The vast majority of injuries that do happen are very minor (e.g., sprains and strains, bumps and bruises) [84,85,86,87,88]. Nevertheless, less minor injuries do occur as a result of normal and healthy active play, and these tend to be fractures to the upper limbs. Two large studies that included all children in a school district (15,074 Canadian students [89]; 25,782 New Zealand students [84]) reported there were no fractures to the head or spine as a result of a fall from playground equipment over the course of the 1 year and 2.5 year assessment periods respectively. Furthermore, findings from a recent systematic review by Nauta and colleagues [85] indicated that while the total number of reported injuries during children’s unstructured play (identified as mainly climbing frames, monkey bars and trampolines) was high compared with sport and active transportation, the incidence rate of medically treated injuries per 1000 hours of unstructured physical activity was lower than for sports and active transportation. At the rate of unstructured play injuries reported by Nauta *et al.* [85] (average rate across all studies presented in the paper was 1.5 injuries per 10,000 hours), if a child were to play for 3 hours a day every day it would take them over 10 years to accumulate 10,000 hours, and presumably 1 or 2 injuries.

“Canadian children are eight times more likely to die as a passenger in a motor vehicle than from being hit by a vehicle when outside on foot or on a bike.”

While parents and other care-providers are worried about letting children walk to places without adult supervision, data from the Public Health Agency of Canada indicate that motor vehicle traffic collisions are the leading cause of death resulting from injuries among Canadians aged 1 to 24 years [90]. Rothman and colleagues [91] mapped the location of child pedestrian collisions in Toronto, and examined the influence of the number of children walking and characteristics of the built environment. They found that after controlling for the built environment, the number of children walking was not associated with the likelihood of collisions. Their results suggest that walking to/from school is relatively safe, even in the City of Toronto, and that safety could be further enhanced by improving the design of intersections [91]. In this regard, an evaluation of the Safe Routes to School program (which has the dual objective of increasing active school transportation and improving safety) in New York City revealed a 44% decrease in pedestrian injuries among children [92].

“There are consequences to keeping children indoors—is it really safer? When children spend more time in front of screens they are more likely to be exposed to cyber-predators and violence, and eat unhealthy snacks.”

The potential use of the Internet for sexual predation is a growing concern for parents, psychologists, and child care providers [93]. In 2012 in Canada, there were 543 cases of luring a child through the Internet that were reported to the police [94]. Moreover, in a large study of youth, victimization by both traditional bullying and cyber-bullying were associated with substance use, violent behavior, unsafe sexual practices, and suicidal behavior [95]. Exposure to cyber-predators and cyber-bullying is obviously minimized by spending less time on the Internet. Browne and Hamilton-Giachritsis [96] have noted that violent imagery on television, video, and computer games is consistently associated with an increase in the likelihood of violent or fearful behavior, particularly among young children. With respect to unhealthy snacks, Borghese and colleagues [40] have reported that television viewing was associated with a lower frequency of consumption of healthy foods (fruits, vegetables, and green vegetables), and a higher frequency of consumption of unhealthy foods (sweets, soft drinks, diet soft drinks, pastries, potato chips, French fries, fruit juices, ice cream, fried foods, and fast food). Such a food consumption pattern may partly explain the consistent relationship between TV time and less favorable body composition among children and youth [76].

“*Air quality indoors is often worse than outdoors, increasing exposure to common allergens (e.g., dust, mould, pet dander), infectious diseases, and potentially leading to chronic conditions.*” 

In an attempt to protect children from traffic-related air pollution and smog, public health authorities may recommend keeping children inside on days where the air quality is poor (e.g., www.peelregion.ca/health/cleanairpeel/smog-health.htm [97]). However, there is evidence showing that air quality may be even worse indoors than outdoors [41], and that exposure to a wide range of substances (e.g., dust mites, cockroaches, domestic pet dander, moulds, fungal spores, nitrogen dioxide, formaldehyde, volatile organic compounds) in the home environment can increase the risk of asthma [42,98]. Furthermore, the World Health Organization [99] estimated that, in 2012, approximately 4.3 million deaths were attributable to household air pollution. These deaths resulted primarily from stroke, ischemic heart disease, chronic obstructive pulmonary disease, acute lower respiratory disease, and lung cancer. 

“*In the long-term, sedentary behaviour and inactivity elevate odds of developing chronic diseases, including heart disease, type-2 diabetes, some forms of cancer and mental health problems.*”

We are often focused on immediate and short term outcomes when it comes to children’s health and well-being, yet the decisions we make during childhood have consequences across the lifespan, and should be considered. Lee *et al*. [100] estimated that physical inactivity causes 6% of the global burden of disease for coronary heart disease, 7% for type-2 diabetes and 10% for both breast and colon cancer. Overall, physical inactivity caused approximately 9% of all deaths worldwide in 2008 [100]. Physical inactivity in childhood/adolescence also leads to increased cardiovascular risk among older adolescents [101] and young adults [102]. Conversely, two very large randomized controlled trials on adults have noted that a healthy active lifestyle (including daily physical activity and healthy eating) reduced the incidence of type-2 diabetes by 58%, even outperforming the commonly-used drug metformin [103,104]. A systematic review of prospective studies also found consistent evidence that physical inactivity elevates the risk of depression [105]. Moreover, large prospective studies have consistently shown lower risk of mortality among more active individuals [106,107,108] and higher risk among more sedentary individuals [109].

“*Hyper-parenting limits physical activity and can harm mental health.*”

Children today have little discretionary time available for self-directed play [18] and hyper-parenting appears on the rise [110]. Janssen [110] examined how 7–12 year olds’ physical activity was influenced by the four following hyper-parenting styles: (1) “helicopter parents” who try to protect their children from all dangers and solve all of their problems; (2) “little emperor” parents who endeavor to satisfy all of their children’s material desires; (3) “tiger moms” who push their children to be exceptional in all domains; and (4) those who practice “concerted cultivation” (e.g., scheduling their children into several extracurricular activities to provide them with an advantage). With the exception of “helicopter parents”, hyper-parenting was associated with significantly lower physical activity. The American Academy of Pediatrics [111] has raised concerns that the substitution of play with academics and enrichment activities may have adverse consequences for children’s mental health. College students who reported having “helicopter” parents reported higher levels of depression and less satisfaction with life [112]. In a study of 317 US college students, helicopter parenting was negatively related to psychological well-being and positively related to prescription medication use for anxiety/depression and the recreational consumption of pain pills [113].

“*When children are closely supervised outside, they are less active.*” 

Children’s independent mobility—their freedom to play and travel around their own neighbourhood without adult supervision—has decreased markedly over the last decades [114,115,116,117], prompting research examining the relationship between independent mobility and physical activity. Recent studies provide consistent evidence that children who have greater independent mobility are significantly more active [118,119,120,121,122,123]. In addition, Floyd and colleagues [124] investigated the factors associated with children’s park-based physical activity using direct observation in a large sample of children. They noted that the presence of a parent or another adult was associated with reduced physical activity. Together, these findings emphasize the importance of unsupervised outdoor activities.

“*Children are more curious about, and interested in, natural spaces than pre-fabricated play structures. Children who engage in active outdoor play in natural environments demonstrate resilience, self-regulation and develop skills for dealing with stress later in life*.”

Adults tend to create outdoor play spaces in line with their perspective of what is attractive to children (e.g., bright colors) with a maximum of safety [125], although children have a preference for natural outdoor play environments [126,127,128,129,130,131]. Settings that are malleable, as is characteristic of many natural elements, afford a greater range of play and are thus more attractive to children [131]. Natural outdoor environments provide opportunities to engage in the most complex forms of play, including active play [130]. Luchs and Fikus [130] compared children’s play behaviours on a nature-based playground with a contemporary playground and found longer and more complex play episodes on the nature-based playground. Nature affordances (e.g., leafs, branches) prompt children’s creative play and imagination [132]. Natural environments allow for playful bodily experiences such as running, tumbling, and being in water that can open up the sense of playfulness, which is so vivid in childhood [126]. In addition, “green exercise” may provide a more enjoyable form of physical activity to children who are not typically active [133]. Herrington *et al*. have also found that natural play spaces provide more opportunities for diverse play activities and developmental gain than pre-fabricated play structures [134,135]. Jones posits the seemingly lucid question “Could it be that within all these pedagogic, economic, legislative, technological and emotional interventions and investments in childhood, society has somehow missed what children really are, and what they really need?” ([125], p. 198).

Hüttenmoser compared 5-year-olds permitted independent mobility with socio-economically matched children whose parents did not allow independent mobility (due to perceived traffic safety concerns) [136]. He found better motor skills, social behaviour, independence and conflict resolution skills in children with ready access to outdoor active play. Prezza *et al*. found similar relationships among children 7–12 years of age [137]. Becker *et al*. observed that higher active outdoor play was associated with better self-regulation and in turn with better academic achievement among pre-school children [138]. Others have shown similar psychological benefits of active outdoor play [139,140,141,142].

Observational studies of children at play outdoors found that they exposed themselves to risk, but displayed clear strategies for mitigating harm [143,144]. Children participating in a 14-week risky play intervention improved their reaction time in detecting risk, increased self-esteem, and decreased conflict sensitivity relative to their pre-intervention performance, and when compared to a control group [145]. Opportunities for some risk in play promote learning of risk management strategies, which are fundamental for children to keep themselves safe in the diverse situations typical in daily life [16,32,44]. Experience with managing risks during childhood has also been associated with the ability to negotiate decisions about substance use, relationships and sexual behaviour during adolescence [16,37].

Gray hypothesized a direct link between generational increases in psychopathology and declines in play time [146]. Current generations of youth have 5-8 times more clinically significant scores on standardized measures of anxiety and depression when compared to youth in the 1950’s [147,148]. Gray cites evidence indicating that play deprivation contributes to a reduced sense of personal control, reduced ability to control emotions, increased social isolation, and reduced happiness [146]. Furthermore, it has been shown that natural environments are sensory-rich environments that contribute to problem-solving skills, self-regulation, and resilience [149,150] and the natural outdoors fosters creative thinking [151]. 

“*Outdoor play that occurs in minimally structured, free and accessible environments facilitates socialization with peers, the community and the environment, reduces feelings of isolation, builds inter-personal skills and facilitates healthy development*.”

The natural outdoors is a minimally structured environment that encourages children to engage in more social interaction with peers [132]. When children engage in outdoor play, they construct meaning in order to satisfy innate curiosity, and learning happens while interacting with peers [126]. Furthermore, in addition to facilitating physical activity, independent mobility enables children to socialize with their peers [115,137,152] and to develop their way-finding skills [153,154]. Independent mobility also fosters the development of children’s global sense of agency and a more positive relationship with the environment [155,156].

### 3.2. Recommendations

Like physical activity in general [157], children’s active outdoor play is affected by multiple levels of influence [158,159]. Therefore, consistent with social-ecological models of health behavior [157,160,161,162], the recommendations included in the position statement (Supplementary 2) target multiple levels of influence including the family, the social environment, the built environment and public policies. In agreement with the Ottawa Charter for Health Promotion [163], the intent is to make the healthy choice the easy choice, and the norm. This could be achieved by: (1) increasing opportunities for children to play outdoors in all settings including their home, school, childcare center, and neighbourhood environments; (2) addressing key barriers to active outdoor play (in particular restrictive design guidelines and the fear of lawsuits related to liability issues); and (3) avoiding exaggeration of risks associated with active outdoor play which may discourage parents from letting their children go outdoors.

### 3.3. Stakeholder Survey Findings

During the two and a half weeks that the on-line stakeholder survey was open, 2514 respondents landed on the front page. Responses varied by question (1702 to 1908 responses for close-ended questions; 558 to 641 responses for open-ended questions) with lower rates on questions in the middle to end of the survey. Respondents were from every province and two of the three territories in Canada with the majority living in Ontario (28%), Alberta (22%), British Columbia (16%) and Quebec (11%). International respondents (2%) were from the United States, the United Kingdom, Ireland, Australia and several countries in mainland Europe and Asia. The majority of respondents identified as health professionals (29%), early childhood care providers (16%), government (15%), teachers (12%) and non-government organizations/board members (10%). 

For all sections of the *Position Statement* (position, context, evidence, recommendations), the proportion of respondents who strongly agreed or somewhat agreed that the sections were clearly stated ranged from 96% to 98%. The proportion who strongly agreed or somewhat agreed with the message in these sections ranged from 97% to 99% (Table 3). From the open-ended questions, the most frequently occurring concerns and suggestions were in relation to the *Position Statement’s* evidence (lack of supporting references—now added), tone (negative and condescending in places) and terminology (e.g., “risk”, “hyper-parenting”, “loosening the reins”). Changes were made accordingly as described in the Methods. 1008 respondents indicated interest in being supporters of the *Position Statement* once it was finalized.

**Table 3:** Stakeholder assessment of the *Position Statement’s* clarity and stakeholder level of agreement, by section.

Section Title	Section Clearly Stated				Agreement with Section			
	Total N	Strongly Agree	Somewhat Agree	Combined Agreement	Total N	Strongly Agree	Somewhat Agree	Combined Agreement
Statement	1903	1439 (75.6%)	410 (21.5%)	1849 (97.1%)	1908	1738 (91.1%)	153 (8.0%)	1891 (99.1%)
Context	1809	1384 (76.5%)	374 (20.7%)	1758 (97.2%)	1811	1575 (87.0%)	201 (11.1%)	1776 (98.1%)
Evidence	1770	1251 (70.7%)	445 (25.1%)	1696 (95.8%)	1773	1438 (81.1%)	284 (16.0%)	1722 (97.1%)
Recommendations	1723	1290 (74.9%)	393 (22.8%)	1683 (97.7%)	1727	1388 (80.4%)	298 (17.3%)	1686 (97.7%)

The launch of the *Position Statement on Active Outdoor Play* and the release of the ParticipACTION Report Card occurred on June 9th, 2015. The Report Card and *Position Statement* as well as related media and stakeholder toolkit materials are available at www.ParticipACTION.com.

## 4. Discussion

The aim of this paper was to provide a thorough and transparent description of the process used to create this evidence-informed *Position Statement on Active Outdoor Play* for use by stakeholders committed to the promotion of healthy active living among children. The multi-sectorial, multi-disciplinary nature of the consensus group, and the extensive stakeholder consultation, were clear strengths of the initiative. Bringing researchers and advocates for physical activity and play in nature and the outdoors together with those from injury prevention, insurance and law created a unique, respectful, and impactful environment where different opinions surfaced and a balanced outcome emerged.

The debate regarding the benefits and harms of active outdoor play has typically pitted injury prevention, insurance and liability concerns against those promoting physical activity and child development. This *Position Statement on Active Outdoor Play* development process has been unique in bringing together professions that have traditionally been at odds to develop an evidence-based position statement that all parties can support. It represents an opportunity to develop a shared national vision that can ensure joint messaging and provide support to agencies across Canada in their efforts to promote active outdoor play locally within their communities. 

While bringing groups with sometimes divergent views together was a strength of this process, it predictably came with its challenges. The inability to completely overcome these challenges is reflected by three participating organizations unable to endorse the final *Position Statement* as presented in the results. While these three groups were very supportive of the overall intent of the *Position Statement*, they felt certain aspects exceeded their comfort zone. One specific recommendation was particularly controversial: “Provincial and Municipal Governments: Work together to create an environment where Public Entities are protected from frivolous lawsuits over minor injuries related to normal and healthy outdoor risky active play. This protection would no longer restrict Public Entities to using the Canadian Standards Association CAN/CSA Z614 “Children’s Playspaces and Equipment” as a guide for the design of outdoor play spaces and as a requirement for the funding of these spaces. An increased investment in natural play spaces in all neighbourhoods is encouraged.” From the comments of the concerned organizations their interpretation of this recommendation is that removing the *requirement* of all outdoor play spaces adhering to this guideline will somehow place children at unacceptable risk. Despite this concern we elected to leave this recommendation in. There are several lines of reasoning for this decision.

1. We believe the CSA Z614 Standards need to be re-examined to ensure that they consider the latest injury data and research on children’s outdoor play and the importance of risky play in children’s health and development. Because currently funding tends to be tied to meeting CSA Standards, we are not convinced that the other side of the healthy development equation (i.e., health benefits of play with an element of risk) are adequately considered in the science and injury statistics used to date. We know very little about how the denominator of “child use hours” is considered when interpreting injury statistics.
2. Of the nearly 2,000 stakeholder survey respondents (a presumably informed sample) 21/1 199 comments provided even mentioned CSA (positive or negative) suggesting that to the larger sector represented by stakeholders sufficiently engaged to not only answer the survey but provide comments is generally supportive of this recommendation.
3. Based on the experience of many on consensus group, the CSA guideline is frequently cited by school and recreation administrators as a barrier to the development of more nature-based play spaces. The CSA Z614 Standards’ focus on structures, equipment, and surfacing materials [164] results in play spaces that are more likely to consist of equipment than natural play environments with loose materials and as the *Position Statement* points out, this may limit children’s interest, enjoyment and participation. The research indicates that play in nature is more complex and diverse than equipment based playgrounds, and is longer in duration [130,131,165,166]. Play in nature also increases moderate-to-vigorous physical activity [167]) and light to moderate physical activity among children [168,169]. Play in nature fosters self-determination [149] and helps children with emotional and behavioural problems [170,171]. Natural play environments are also more gender neutral and offer more gender equity [172].
4. From the consensus group deliberations, the comments received in the stakeholder survey, and the evidence supporting natural play spaces for children, there is ample support to consider alternate options to the CAN/CSA Z614 guideline. We are not recommending the elimination of the guideline, but rather revision or allowance of alternate approaches. For example, the guideline could be revised to better accommodate natural play spaces; the guidelines could allow for other guidelines to apply; or a different approach (e.g., Play Safety Forum: Managing Risk in Play Provision [173]) could be employed.
5. Finally, a very engaged youth group submitted the following quote after in-depth discussions on the *Position Statement*: “From our experience as Canadian young people, this statement coincides with what we believe contributes to fun, healthy, and active child development. From our perspective, which stems from our research and personal experience, this statement accurately identifies priorities for child active outdoor play.” (Child health 2.0 Youth Advisory Board and Child health 2.0 Research Team). While it could be argued that this group is not intimately familiar with CSA guidelines, their resounding support provides a further layer of reassurance that the core intent of the *Position Statement* is aligned with what youth want.

Play is an important childhood need, so fundamental it is enshrined in the United Nations Convention on the Rights of the Child [11,174]. The accompanying systematic reviews to this paper highlight the particular importance of active outdoor play for children’s health and development [43,46]. Yet recent decades have seen a trend toward greater monitoring and restrictions on children’s play, and currently half of Canadian children play actively outdoors for fewer than 3 hours per week [18]. International child health experts have expressed concern over this low level of active outdoor play. The U.S. Centers for Disease Control Obesity Center Director wrote that opportunities for play may be the “only requirement” for increasing children’s physical activity [175]. Similarly, Burdette & Whitaker argued that play can *“improve all aspects of children’s well-being: physical, emotional, social, and cognitive*” [176].

The UK’s Play Safety Forum’s [36] 2008 position statement encouraging provision of risky play was endorsed inter alia by the UK government’s Health and Safety Executive and facilitated shifting their approach to play provision [177]. To our knowledge, this *Position Statement* is the first to fully disclose, document, and have peer-reviewed the process undertaken and the outcomes achieved. With our broad sector representation, the rigour of the *Position Statement* development process, and accompanying national social marketing campaign, we are aiming to shift Canadians’ approach towards and anxiety around children’s active outdoor play with targeted messaging for all relevant stakeholders. Campaigns like this have been successful in influencing policies and practices in the past [60].

Parental and societal anxieties, attitudes about social dangers, and perceptions of the value of free play and outdoor autonomy exert a strong influence on children’s outdoor risky play opportunities [16,27,81,158,178,179,180,181,182]. A shift in perceptions of risk is necessary, changing from a perspective of danger and harm to one of challenge, adventure and opportunity. Previous work such as Nieheus *et al.*’s Opportunity for Adventure risk reframing intervention, has suggested that such a shift is possible, [183,184,185]. They describe several ‘aha’ moments whereby intervention participants realize the potential negative impacts of having a fearful approach to children’s risky play and the benefits of supporting children’s health risk taking. For example, parents (all mothers) commonly expressed wanting to raise independent children, and through the session realizing that their own anxiety-based care-giving was limiting their children’s opportunities for the new experiences and challenges necessary for self-sufficiency. Interventions to encourage outdoor risky play opportunities may particularly resonate for fathers, who are more likely than mothers to engage in risky play and endorse risk taking as important for facilitating children’s physical development and self-confidence [32,186,187,188]. Research could incorporate instruments such as the Tolerance of Risk in Play Scale (TRiPS), to assess differences or changes in attitudes [189]. 

Recent evidence has also shown that risk-reframing (less fear and greater appreciation for risky play) can be successfully achieved through a training intervention in early childhood pre-service teachers [190]. Also, the theoretical underpinning of early childhood pedagogy may be important in the alignment of early childhood teachers’ beliefs and practices relating to risky outdoor play. An early childhood teacher education that focuses on theories on the individual’s interrelationship with the environment through perception and utilization of affordances, where children’s learning is put in direct relation to their actions in various environments, coupled with a strong emphasis on early childhood teachers’ own practical skills of physical active and outdoor play seems to enhance children’s opportunity of active outdoor play in child care institutions [191].

The release of the *Position Statement* will benefit greatly from the proactive distribution, public relations and media strategy prepared to maximize the reach and impact of the release of the ParticipACTION Report Card [60]. Through the Report Card, the *Position Statement* will directly reach relevant government and non-government organizations whose work touches child and youth physical activity across Canada. The general public, parents, and youth will also be reached indirectly through the media and directly through public-facing communications channels (social media, blog, website) of ParticipACTION and collaborating organizations (see author list and affiliations). To reach the audiences specifically identified within the *Position Statement,* it will be further shared by the collaborating organizations through their networks. Post release, evaluation will occur as part of the larger Report Card evaluation plan, including tracking of distribution, media impressions, hits and quality, website traffic, and social media reach and engagement among Canadians. Surveys of Report Card users and debrief meetings with ParticipACTION’s strategic partners and collaborating individuals and organizations will provide insight into the perceived impact of the *Position Statement,* and report Card, within each province and territory.

In addition to dissemination and evaluation plans, efforts to ignite action to advance the advice contained in the *Position Statement* were initiated during the development process. For example, the Active Canada 20/20 Active Play Action Group began soliciting funding to develop a supportive tool-kit to implement recommendations contained in the *Position Statement* before it was even released. Similar coordinated and proactive planning was done by The Lawson Foundation and ParticipACTION. This proactive coordination should help give life to the *Position Statement* in a timely fashion.

While this paper and the accompanying systematic reviews [43,46] provide substantial evidence supporting our *Position Statement*, there are many future research needs; a list of research priorities is provided below.

- Future research should further compare the benefits and harms between active play in natural environments and other outdoor environments, as current evidence is mixed. For instance, Coe *et al.* [192] found that children (aged 3–5 years) were more active in natural environments, while another study indicated that both natural and traditional playgrounds interventions can increase the time spent in moderate-to-vigorous physical activity, but the more traditional playground interventions were more effective at increasing physical activity in children (aged 8–9 years)[192].
- Future research should also evaluate promising approaches to risk reframing, especially regarding the perceptions that society has on the role of women who are responsible for the care of children. Despite societal changes, mothers tend to remain responsible for the care of children and the accompanying risk management. This responsibility felt by mothers, influences their children’s outdoor play [193].
- Research that concurrently considers the benefits and harms of active outdoor play is required, including exploring age and gender-related differences. Research tends to study the benefits and harms in isolation rather than looking at the overall health and well-being of the child.
- Further research that investigates if engagement in active outdoor play and risky play during the childhood years offers some protection against unintentional injuries during the adolescent and adult years is required. For example, what risk management skills are gained by these experiences and how do they influence the individual’s ability to navigate risks in different environments and circumstances both short- and long-term?
- Further research is needed to assess the influence of active outdoor play and risky play on children’s risk management. A 14-week risky play intervention that showed improved reaction time in detecting risk [145] provides emerging evidence. Additional research should explore the influence on executive functioning and on real-world risk decision-making.
- Injury surveillance that includes measures of exposure would provide a more accurate sense of the likelihood of serious injury while engaged in active outdoor play. Nauta *et al*.’s [85] systematic review indicated lower injury rates for unstructured play, compared to sports and active transportation, when the magnitude of exposure was considered. Consistency in measurement strategies and definitions of serious injury would also improve estimates.
- Additional research on the barriers and enablers for parents, teachers, care-providers and policy-makers to promote and facilitate active outdoor play that is required to inform and improve future interventions.
- More evidence from stronger research designs (e.g., randomized controlled trials) with valid and reliable measures is needed.

## 5. Conclusions

There is broad support from the published literature, the consensus group, and the stakeholders consulted that the optimal balance between health promotion through active outdoor and risky play, and injury prevention and safety concerns, has been lost for children today. The *Position Statement on Active Outdoor Play* presented here is intended to facilitate a recalibration of attitudes, practices, policies, and ultimately normative behaviors to promote healthy child growth and development. 

## Supplementary Files

Supplementary File 1

Supplementary File 1
