# Supplementary material for: Position Statement on Active Outdoor Play
Source: Int J Environ Res Public Health. 2015 Jun 8;12(6):6475–505. doi: 10.3390/ijerph120606475 (PMC4483712; doi:10.3390/ijerph120606475)
Supplement: Supplementary File 1 [file ijerph-12-06475-s001.pdf]

Supplementary Information

A Position Statement on Active Outdoor Play

Investigators: Dr. Mark Tremblay, Healthy Active Living and Obesity Research Group, Children's Hospital of Eastern Ontario (CHEO), email: mtremblay@cheo.on.ca; Dr. Casey Gray, Healthy Active Living and Obesity Research Group, Children's Hospital of Eastern Ontario (CHEO), email: casgray@cheo.on.ca. You are being invited to participate in a survey soliciting your opinion on the draft Position Statement on Active Outdoor Play, prepared by a group of 17 concerned Canadian organizations. You are being invited to participate because you are a practitioner whose work is in some way connected with children's outdoor active play. In the past few decades, the prevalence of obesity, physical inactivity and sedentary behaviors among children in Canada has risen, and children are spending more time indoors and less time outdoors and with nature. A recent systematic review completed by our group uncovered strong evidence that children who spend more time outdoors have higher physical activity and lower sedentary behavior levels. A second systematic review completed by our group showed that various forms of active outdoor play (e.g., independent mobility) are related to increased physical activity, decreased sedentary behavior, and improved social outcomes in children. Despite these benefits of outdoor active and even risky play, the potential harms perceived to be associated with increased time in nature and the outdoors appear to be swinging the pendulum to a point where healthy child development may be at risk—ironically in an attempt to actually reduce risk. Related to this, we brought together previously fractious/dissenting groups in the health promotion community to work constructively together for the promotion of childhood health and wellness. As a result, a group of 21 Canadian experts, representing 17 organizations, convened to examine the best available evidence on outdoor and risky active play and develop an evidence-informed position statement. This Position Statement outlines the evidence, and provides our recommendations for action. As the final stage in the development of this Position Statement we are seeking to gain feedback about the clarity of the Position Statement, as well as level of agreement, perceived importance, and support for the Position Statement from a large number of practitioners in the fields represented by each of the contributing collaborators (e.g., parks, green cities, pediatricians, physical activity). We encourage you to circulate the survey link to your colleagues and among your networks. This stakeholder survey will be open until March 15th, 2015. The survey should take you no longer than 10 minutes to complete. On the next series of screens you will be presented with each proposed section of the Position Statement. You may advance or go back using the buttons on the bottom of the screen. At any time during the survey, you may switch between French and English versions of the survey by using the language option in the drop-down menu at the top of each page. But if you are entering data on a page, make sure you save the data by clicking on the "Next" button before using the language option. Participation in this survey is voluntary. By accessing and completing this survey you are giving your implied/passive consent to participate in the survey. The survey does not collect information about your name or email address and responses will be presented in group format only. A summary of findings will be available on the HALO website (www.haloresearch.ca) once the results have been published and will also be sent to participating organizations for distribution to members. If you have any questions about this study, please contact

Dr. Mark Tremblay at 613-737-7600 ext 4114 or mtremblay@cheo.on.ca. The CHEO Research Ethics Board (REB) has reviewed this protocol. The REB considers ethical aspects of all research studies involving human participants at the CHEO and its Research Institute. If you have any questions about your rights as a study participant, you may contact the CHEO REB Chairperson at 613-737-7600 ext 3624.Note: This Position Statement on Active Outdoor Play is in draft form and not intended for general circulation.
Version 3_February 24, 2015

PAGE 1—Position

Access to active play in nature and outdoors - with its risks - is essential for healthy child development. We recommend increasing children's opportunities for self-directed play outdoors in all settings - at home, at school, in child care, in the community and in natural environments.

1. Is the Position clearly stated?

○ Strongly Agree

○ Somewhat Agree

○ Neither Agree Nor Disagree

○ Somewhat Disagree

○ Strongly Disagree

2. Do you agree with the Position?

○ Strongly Agree

○ Somewhat Agree

○ Neither Agree Nor Disagree

○ Somewhat Disagree

○ Strongly Disagree

PAGE 2—Context

In an era of schoolyard ball bans and debates about safe tobogganing, have we lost the appropriate balance between keeping our kids healthy and active and protecting them from serious harm? If we hover over our kids and make too many rules about what they can and can't do, will we hinder their natural ability to develop and learn? If we modify playgrounds in an attempt to prevent all injuries - and lawsuits –will we take the fun away? Is it really safer for our kids to be sitting still on the couch instead of playing actively outside? We need a better balance between perceived danger, real danger and acceptable risk. And we need a better balance between short-term safety and long-term health. Risk is often seen as a bad word - by parents, neighbours, care providers, insurance policy-holders, schools and municipalities. But "risky active play" doesn't mean courting danger or being

unsafe, like skating on a half-frozen lake. It means giving kids the freedom to learn to climb up high, explore the woods, get dirty, play hide 'n seek, wander in their neighbourhoods, balance, tumble and rough-house, especially outdoors, so they can be active, build confidence, autonomy and resilience, develop skills, solve problems and learn their own limits. It’s letting kids be kids - healthier, more active kids.

3. Is the Context clearly stated?

- ○ Strongly Agree
- ○ Somewhat Agree
- ○ Neither Agree Nor Disagree
- ○ Somewhat Disagree
- ○ Strongly Disagree

4. Do you agree with the Context?

- ○ Strongly Agree
- ○ Somewhat Agree
- ○ Neither Agree Nor Disagree
- ○ Somewhat Disagree
- ○ Strongly Disagree

PAGE 3—Evidence

When kids are outside they move more, sit less, and play longer - plus they get access to fresh air and vitamin D. The perception that outdoor play is dangerous is incorrect. The odds of total stranger abduction are about 1 in 14 million based on RCMP reports. Being with friends outdoors may further reduce this risk. Broken bones and head injuries unfortunately do happen, but major trauma is uncommon. Most injuries associated with outdoor play are minor. Canadian children are eight times more likely to die as a passenger in a motor vehicle than from being hit by a vehicle when outside on foot or on a bike. There are risks involved with keeping kids indoors. When kids spend more time in front of screens they are more likely to be exposed to cyber-predators and violence and eat unhealthy snacks. Air quality indoors is often worse than outdoors, increasing exposure to common allergens (e.g., dust, mould, pet dander) and infectious diseases. In the long-term, sedentary behaviour and inactivity elevate risks for chronic disease, including heart disease, type-2 diabetes, some forms of cancer and mental health problems. In many circumstances hyper-parenting does more harm than good. When children are closely supervised, they are less active. Kids are more curious about, and interested in, natural spaces than pre-fabricated play structures. Kids who play in natural environments demonstrate resilience, self-regulation and develop skills for dealing with stress later in life. Outdoor play that occurs in minimally structured, free and accessible environments facilitates socialization with

peers, the community and the environment, reduces feelings of isolation, builds inter-personal skills and facilitates healthy development.

5. Is the Evidence clearly stated?

- ○ Strongly Agree
- ○ Somewhat Agree
- ○ Neither Agree Nor Disagree
- ○ Somewhat Disagree
- ○ Strongly Disagree

6. Do you agree with the Evidence?

- ○ Strongly Agree
- ○ Somewhat Agree
- ○ Neither Agree Nor Disagree
- ○ Somewhat Disagree
- ○ Strongly Disagree

PAGE 4—Recommendations

Parents: Loosen the reins on your kids, children want and need to engage with their environments more fully. When children are supported to take risks, they are often more engaged, and learn how to assess and manage risk in all areas of their lives. Educators and Caregivers: Regularly embrace the outdoors for learning, socialization and physical activity opportunities, in a variety of weather conditions. Recognize that risky active play is an important part of childhood and should not be eliminated from the school yard or childcare centre. Health Professionals: Be influential! Promote every child's connection with nature and the outdoors - identify outdoor play resources and partner with municipalities, parks, nature-related organizations, parent groups and children to support this process. Injury Control Professionals: Recognize the long-term dangers of a sedentary lifestyle, and not just the acute potential for risks – find a balanced approach to health promotion and protection. School and Child Care Administrators: Choose natural elements over pre-fabricated playgrounds and paved areas - and encourage children to play in, and help design, these environments. Media: Provide balanced reporting - avoid sensationalizing stories about predators and danger; and cover success stories related to outdoor and risky active play. Attorneys General: Establish reasonable liability limits for municipal governments – this means Joint and Several Liability Reform. Provincial and Municipal Governments: Work together to create an environment where Public Entities are protected from frivolous lawsuits over minor injuries related to normal and healthy outdoor risky active play. This protection would no longer restrict Public Entities to using the Canadian Standards Association CAN/CSA Z614 "Children's Playspaces and Equipment" as a guide for the design of outdoor play

spaces. Stop linking this standard to the successful funding of these spaces; invest in natural play spaces in all neighbourhoods. Municipalities: Examine existing by-laws and reconsider those that pose a barrier to active outdoor play. Federal and Provincial/Territorial Governments: Collaborate across sectors to find ways to improve children's access to risky active play in nature and the outdoors. Society: Recognize that kids are competent and capable, and increase their opportunities for self-directed play in nature and the outdoors. Allow all children to play with and form a lasting relationship with nature on their own terms – their health, and the health of the planet, will be better for it!

7. Are the Recommendations clearly stated?

○ Strongly Agree

○ Somewhat Agree

○ Neither Agree Nor Disagree

○ Somewhat Disagree

○ Strongly Disagree

8. Do you agree with the Recommendations?

○ Strongly Agree

○ Somewhat Agree

○ Neither Agree Nor Disagree

○ Somewhat Disagree

○ Strongly Disagree

9. Do you have additional suggestions or hesitations regarding the Recommendations?

PAGE 5—Other Feedback

10. Is this Position Statement on Active Outdoor Play important to public health?

○ Yes

○ No

11. Is this Position Statement on Active Outdoor Play important to you and/or your job?

○ Yes

○ No

12. In the box below, please enter any comments that you would like to add regarding this Position Statement on Active Outdoor Play.

☐

PAGE 6 Demographic Information

13. What area(s) of practice do you represent?

- ☐ Early childhood care provider
- ☐ Teacher
- ☐ Paediatrician
- ☐ Physician
- ☐ Other Health Professional (e.g., public health, health promotion, physiotherapist, nurse, dietitian)
- ☐ Fitness professional (e.g., CSEP-CPT, CSEP-CEP, kinesiologist)
- ☐ Injury prevention
- ☐ Insurance
- ☐ Law
- ☐ Professor/researcher
- ☐ NGO staff or Board Member
- ☐ Government
- ☐ Other, please specify... ____________________

14. Are you a parent or regular caregiver (babysitter, home daycare, etc.) for a child aged 3 to 12 years?

- ○ Yes
- ○ No

15. Where do you primarily live / practice?

- ○ Alberta
- ○ British Columbia
- ○ Manitoba
- ○ New Brunswick
- ○ Newfoundland and Labrador
- ○ Northwest Territories
- ○ Nova Scotia

- ○ Nunavut
- ○ Ontario
- ○ Prince Edward Island
- ○ Quebec
- ○ Saskatchewan
- ○ Yukon Territory
- ○ Outside Canada
- ○ If you selected outside Canada, please indicate country... ____________________

16. When the final version of the Position Statement on Outdoor Active Play is complete, would you like to be contacted for final review so that if supportive, you can decide if you would like to be listed in a "supported by" section associated with the Position Statement?

- ○ Yes
- ○ No
- ○ Don't Know

17. If you answered "Yes" to the previous question, please provide your email address so we can send you the final version (in approximately 6 weeks) and if supportive, gather your name, how you would like to be identified, and province/country to be listed in a "supported by" section associated with the Position Statement.

In French

Un Énoncé de Position Sur le Jeu Actif à L'extérieur

Titre de l'étude : Sondage d'opinion au sujet d'un énoncé de position sur le jeu actif à l'extérieur Chercheurs : Mark Tremblay (PhD), Groupe de recherche sur les saines habitudes de vie et l'obésité, Centre hospitalier pour enfants de l'est de l'Ontario (CHEO), courriel : mtremblay@cheo.on.ca Casey Gray (PhD), Groupe de recherche sur les saines habitudes de vie et l'obésité, Centre hospitalier pour enfants de l'est de l'Ontario (CHEO), courriel : casgray@cheo.on.ca Vous êtes invités à participer à un sondage sur la version préliminaire d'un énoncé de position sur le jeu actif à l'extérieur, développé par un groupe dont les membres représentent 17 organisations canadiennes préoccupées par le sujet. Vous êtes invités à participer parce que votre travail est, d'une façon ou d'une autre, relié au jeu actif à l'extérieur chez l'enfant. Au cours des dernières décennies, la prévalence de l'obésité, de l'inactivité physique et des comportements sédentaires chez les enfants au Canada a subi une augmentation. Les enfants passent aussi plus de temps à l'intérieur, et moins de

temps à l’extérieur et dans la nature. Une revue systématique de la littérature conduite récemment par notre groupe a relevé des preuves solides au sujet du fait que les enfants qui passent plus de temps dehors présentent des niveaux plus élevés d’activité physique et des niveaux plus faibles de comportements sédentaires. Une seconde revue systématique réalisée par notre groupe a montré que diverses caractéristiques de jeu actif à l’extérieur comportant un risque (p. ex., la liberté de déplacement) sont associées, chez les enfants, à une augmentation de l’activité physique, à une diminution du comportement sédentaire et à des retombées positives au niveau social. Néanmoins, les dangers potentiels perçus associés aux activités à l’extérieur et dans la nature semblent contrebalancer ces bienfaits au point où le développement sain de l’enfant pourrait être compromis, ironiquement, alors que l’on tente de réduire le risque associé au jeu. À ce propos, nous avons invité des groupes œuvrant en promotion de la santé à qui il arrive d'avoir des perspectives discordantes / contrastées, et ce afin qu'ils travaillent ensemble de façon constructive pour la promotion de la santé et du bien-être de l’enfant. Un groupe de 21 experts canadiens, représentant 17 organisations, se sont donc réunis pour examiner les études les plus probantes sur le jeu actif à l’extérieur et le jeu actif comportant un risque pour développer un énoncé de position éclairé. Cet énoncé de position présente les preuves scientifiques et nos recommandations. À cette étape finale du développement de l’énoncé de position, nous cherchons à obtenir les réactions de la part d’un grand nombre de professionnels des domaines représentés par nos collaborateurs (p. ex. parcs, villes vertes, pédiatres, activité physique) sur la clarté de l'énoncé de position, de même que sur le degré d'accord, l'importance perçue et le soutien à son égard. Nous vous encourageons à faire circuler le lien de ce sondage à vos collègues et à travers vos réseaux. Ce sondage sera ouvert jusqu’au le 15 mars 2015 et ne devrait pas vous prendre plus de 10 minutes à remplir. À partir de la page suivante, vous pourrez lire chaque section proposée de l’énoncé de position. Utilisez les boutons au bas de la page pour aller à la page suivante ou à la page précédente. Vous pouvez passer de la version anglaise à la version française du sondage à n’importe quel moment en indiquant votre choix à l’aide du menu déroulant de l’option « langue » située en haut de chaque page. Si vous avez entré une information sur une page, assurez-vous de bien enregistrer cette information en appuyant sur le bouton « page suivante » avant de changer la langue. La participation à ce sondage est tout à fait volontaire. En accédant à ce sondage et en le remplissant, vous donnez votre consentement implicite/passif à y participer. Nous ne vous demanderons pas votre nom ni votre adresse courriel à travers ce sondage et les réponses ne seront présentées que sous une forme regroupée. Un résumé des résultats sera disponible sur le site web du groupe de recherche HALO (www.haloresearch.ca – en anglais seulement) une fois publié et sera aussi envoyé aux organisations participantes pour distribution à leurs membres. Veuillez communiquer avec Mark Tremblay au 613 737-7600, poste 4114, pour toute question au sujet de cette étude. Le comité d’éthique de la recherche (CER) du CHEO a révisé ce protocole. Le CER étudie les aspects éthiques de toute recherche qui implique les sujets humains au CHEO et à son institut de recherche. Si vous avez des questions au sujet de vos droits en tant que participant à l’étude, veuillez communiquer avec le président du CER du CHEO au 613 737-7600, poste 3624. Note : Cet énoncé de position au sujet du jeu actif à l’extérieur est présenté en version préliminaire et ne doit pas être distribué.Version 3_Février 24, 2015

Page 1 - Position

L'accès au jeu actif à l'extérieur et dans la nature, avec les risques que cela comporte, est essentiel au développement sain de l'enfant. Nous recommandons d'augmenter les occasions que les enfants ont de jouer de façon autonome dehors, dans des environnements variés, soit à la maison, à l'école, au service de garde, dans la communauté et dans des environnements naturels.

1. La position est-elle clairement définie?

○ Fortement d'accord

○ D'accord

○ Ni en accord ni en désaccord

○ En désaccord

○ Fortement en désaccord

2. Êtes-vous d'accord avec la position?

○ Fortement d'accord

○ D'accord

○ Ni en accord ni en désaccord

○ En désaccord

○ Fortement en désaccord

Page 2 - Mise en Contexte

La tendance actuelle d'interdire des activités à l'extérieur qui comportent un potentiel de blessures, comme jouer au ballon dans les cours d'école, ainsi que le débat qui entoure la sécurité de la glissade en traîneau, amène à se questionner sur l'équilibre nécessaire entre maintenir nos enfants actifs et en santé, et les protéger de blessures sérieuses. Si nous couvons constamment nos enfants et élaborons trop de règlements sur ce qu'ils peuvent et ne peuvent pas faire, brimons-nous leur capacité naturelle à apprendre et à se développer? Si nous modifions les aires de jeux dans l'objectif de prévenir toutes les blessures et toutes les poursuites judiciaires, en évacuons-nous tout le plaisir? Est-ce vraiment plus sécuritaire pour nos enfants de passer des heures assis sur un fauteuil plutôt que de jouer activement dehors? Nous devons atteindre un meilleur équilibre entre le danger perçu, le danger réel et le risque acceptable. Nous devons aussi atteindre un meilleur équilibre entre la sécurité à court terme et la santé à long terme. Le mot « risque » a mauvaise presse auprès des parents, du voisinage, des professionnels de la santé, des détenteurs de polices d'assurance, des écoles et des municipalités. Toutefois, faire un « jeu actif comportant un risque » ne signifie pas de s'exposer au danger, comme patiner sur un lac semi-gelé, par exemple. Cela signifie plutôt de donner aux enfants la liberté d'apprendre à grimper, d'explorer la forêt, de se salir, de jouer à la cachette, de se promener dans le quartier, de se tenir en

équilibre, de faire des culbutes et de se chamailler, surtout dehors, afin d'être physiquement actifs, de développer diverses habiletés, de même que leur confiance en eux, leur autonomie, leur ténacité, leur capacité à résoudre des problèmes, ainsi que de connaître leurs propres limites. Il s'agit de laisser les enfants être des enfants, mais plus en santé et plus actifs.

3. La mise en contexte est-elle clairement définie?

○ Fortement d'accord

○ D'accord

○ Ni en accord ni en désaccord

○ En désaccord

○ Fortement en désaccord

4. Êtes-vous d'accord avec la mise en contexte?

○ Fortement d'accord

○ D'accord

○ Ni en accord ni en désaccord

○ En désaccord

○ Fortement en désaccord

Page 3 - Preuves Scientifiques

Quand les enfants sont dehors, ils bougent plus, s'assoient moins et jouent plus longtemps. De plus, ils ont accès à de l'air frais et à de la vitamine D. La perception que le jeu à l'extérieur est dangereux est erronée. Les probabilités de se faire enlever par un inconnu sont de 1 sur 14 millions, selon les rapports de la GRC. Être dehors avec des amis pourrait réduire davantage ce risque. Les fractures et les blessures à la tête surviennent malheureusement, mais les traumatismes majeurs sont rares. La plupart des blessures associées au jeu à l'extérieur sont mineures. Les enfants canadiens présentent huit fois plus de risque de mourir en étant passager d'un véhicule motorisé qu'en se faisant heurter par un véhicule lorsqu'ils sont à pied ou à vélo. Il existe des risques à garder les enfants à l'intérieur. Quand les enfants passent plus de temps devant les écrans, ils sont plus susceptibles d'être exposés à des cyberprédateurs, à de la violence et à manger des collations malsaines. L'air est souvent de moins bonne qualité à l'intérieur qu'à l'extérieur, ce qui augmente l'exposition aux allergènes communs (i.e., poussière, moisissures, animaux de compagnie) et aux maladies infectieuses. À long terme, les comportements sédentaires et l'inactivité physique augmentent le risque de maladies chroniques, incluant les maladies cardiaques, le diabète de type 2, quelques formes de cancer et des problèmes de santé mentale. Dans plusieurs circonstances, l'hyper-parentalité cause plus de tort que de bien. Quand les enfants sont supervisés de près, ils sont moins actifs. Les enfants démontrent un plus grand intérêt pour les espaces naturels, plutôt que pour les structures de jeux préfabriquées. Les enfants qui jouent

dans des environnements naturels démontrent de la résilience, du contrôle de soi et développent des habiletés qui les aideront à réagir de façon appropriée au stress au cours de leur vie. Le jeu à l'extérieur qui a lieu dans des environnements minimalement structurés où les enfants peuvent jouer librement favorise les interactions avec l'environnement, la socialisation avec les pairs et avec la communauté, réduit le sentiment d'isolement, développe des habiletés interpersonnelles et favorise un développement sain.

5. Les preuves scientifiques sont-elles clairement définies?

○ Fortement d'accord

○ D'accord

○ Ni en accord ni en désaccord

○ En désaccord

○ Fortement en désaccord

6. Êtes-vous d'accord avec les preuves scientifiques?

○ Fortement d'accord

○ D'accord

○ Ni en accord ni en désaccord

○ En désaccord

○ Fortement en désaccord

Page 4 - Recommandations

Parents : Accordez plus de liberté à vos enfants. Les enfants veulent et ont besoin d'être en relation plus étroite avec leur environnement. Quand les enfants sont soutenus dans leur prise de risques, ils se sentent souvent plus concernés et apprennent à se responsabiliser, à évaluer et à gérer le risque à travers les divers aspects de leur vie. Éducateurs / Gardiens : Profitez régulièrement de toutes les occasions d'aller dehors pour apprendre, socialiser et faire de l'activité physique, et ce, dans une variété de conditions climatiques. Reconnaissez que le jeu actif comportant un risque est un aspect important de l'enfance qui ne devrait pas être éliminé de la cour d'école ou du service de garde. Professionnels de la santé : Profitez de votre influence! Encouragez chaque enfant à développer une relation avec la nature et l'extérieur. Identifiez les ressources pour jouer dehors et développez des partenariats avec les municipalités, les parcs, les organismes qui favorisent le contact avec la nature, les groupes de parents et les enfants eux-mêmes afin de soutenir ce processus. Autorités de santé publique : Reconnaissez les dangers à long terme du mode de vie sédentaire, et pas seulement le potentiel de risque à court terme. Trouvez une approche équilibrée entre la promotion de la santé et la protection. Administrateurs scolaires / de services de garde : Choisissez des éléments naturels plutôt que des structures de jeu préfabriquées ou des surfaces pavées. Encouragez les enfants à jouer, mais

aussi à aider à concevoir ces environnements. Médias : Offrez une couverture équilibrée des événements. Évitez les histoires sensationnalistes au sujet des prédateurs et du danger. Rapportez les histoires à succès sur les bénéfices d'exposer les jeunes au jeu actif à l'extérieur et au jeu actif comportant un risque. Procureurs généraux : Établissez des limites raisonnables de responsabilité pour les autorités municipales qui se traduisent par une réforme de la responsabilité conjointe et solidaire. Gouvernements provinciaux et autorités municipales : Travaillez ensemble pour créer un environnement où les entités publiques sont protégées des poursuites frivoles pour des blessures mineures reliées au jeu actif à l'extérieur et au jeu actif comportant un risque normal et sain. Cette protection ne limiterait plus les entités publiques à utiliser la norme de l'Association canadienne de normalisation CAN/CSA Z614 « Aires et équipement de jeu pour enfants » comme guide pour la conception des espaces de jeux extérieurs. Cessez de relier le respect de ce critère au financement de ces espaces. Investissez dans des aires naturelles de jeux dans tous les quartiers. Municipalités : Révisez les règlements actuels et reconsidérez ceux qui représentent un obstacle au jeu actif à l'extérieur. Gouvernements fédéral / provinciaux / territoriaux : Favorisez la collaboration intersectorielle afin de trouver des façons d'améliorer l'accès des enfants au jeu actif à l'extérieur et dans la nature, et au jeu actif comportant un risque. Société : Reconnaissez que les enfants possèdent des compétences et des habiletés et augmentez les occasions de jeu autodirigé dehors et dans la nature. Permettez à tous les enfants de jouer et de développer une relation durable avec la nature comme bon leur semble. Leur santé et celle de la planète s'en porteront bien mieux!

7. Les recommandations sont-elles clairement définies?

○ Fortement d'accord

○ D'accord

○ Ni en accord ni en désaccord

○ En désaccord

○ Fortement en désaccord

8. Êtes-vous d'accord avec les recommandations?

○ Fortement d'accord

○ D'accord

○ Ni en accord ni en désaccord

○ En désaccord

○ Fortement en désaccord

9. Avez-vous des suggestions additionnelles ou des hésitations au sujet des recommandations?

[ ]

PAGE 5 - AUTRES RÉACTIONS

10. Cet énoncé de position sur le jeu actif à l'extérieur est-il important pour la santé publique?

○ Oui

○ Non

11. Cet énoncé de position sur le jeu actif à l'extérieur est-il important pour vous et/ou pour votre travail?

○ Oui

○ Non

12. Veuillez inscrire tout commentaire additionnel sur cet énoncé de position au sujet du jeu actif à l'extérieur dans la boîte ci-dessous.

PAGE 6 RENSEIGNEMENTS DÉMOGRAPHIQUES

13. Quel(s) domaine(s) de pratique représentez-vous ?

☐ Professionnel en services à la petite enfance

☐ Enseignant

☐ Pédiatre

☐ Médecin

☐ Autre professionnel de la santé (p. ex. santé publique, promotion de la santé, physiothérapeute, infirmière, nutritionniste)

☐ Professionnel de l'activité physique (p. ex. EPC-SCPE, PEC-SCPE, kinésiologue)

☐ Prévention des blessures

☐ Assurances

☐ Droit

☐ Professeur/chercheur

☐ Employé ou membre du conseil d'administration d'un organisme non gouvernemental (ONG)

☐ Gouvernement

☐ Autre, veuillez préciser ____________________

14. Êtes-vous le parent ou prenez-vous soin régulièrement (gardien, éducateur en garderie, etc.) d'un enfant âgé de 3 à 12 ans?

- ○ Oui
- ○ Non

15. Quel est votre principal lieu de résidence/de travail?

- ○ Alberta
- ○ Colombie-Britannique
- ○ Manitoba
- ○ Nouveau-Brunswick
- ○ Terre-Neuve et Labrador
- ○ Territoires du Nord-Ouest
- ○ Nouvelle-Écosse
- ○ Nunavut
- ○ Ontario
- ○ Île-du-Prince-Édouard
- ○ Québec
- ○ Saskatchewan
- ○ Yukon
- ○ Hors Canada
- ○ Si vous avez choisi « hors Canada », veuillez inscrire le pays ____________________

16. Quand la version finale de l'énoncé de position sur le jeu actif à l'extérieur sera terminée, voudriez-vous que l'on communique avec vous pour une révision finale? Si vous l'appuyez, vous pourrez décider si vous souhaitez apparaître dans la section « appui » associée à l'énoncé de position.

- ○ Oui
- ○ Non
- ○ Ne sais pas

17. Si vous avez répondu « oui » à la question précédente, veuillez fournir votre adresse courriel pour que nous puissions vous envoyer la version finale (dans 6 semaines environ). Si vous appuyez toujours l'énoncé de position, nous recueillerons votre nom, la façon dont vous souhaitez être identifié et votre province/pays.

© 2015 by the authors; licensee MDPI, Basel, Switzerland. This article is an open access article distributed under the terms and conditions of the Creative Commons Attribution license (http://creativecommons.org/licenses/by/4.0/).
