# Supplementary material for: Position Statement on Active Outdoor Play
Source: Int J Environ Res Public Health. 2015 Jun 8;12(6):6475–505. doi: 10.3390/ijerph120606475 (PMC4483712; doi:10.3390/ijerph120606475)
Supplement: Supplementary File 1 [file ijerph-12-06475-s002.pdf]

Position

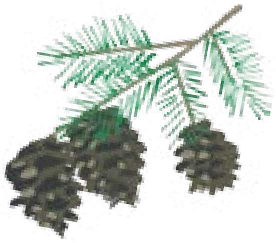

Access to active play in nature and outdoors—with its risks—is essential for healthy child development. We recommend increasing children's opportunities for self-directed play outdoors in all settings—at home, at school, in child care, the community and nature.

PREAMBLE

We conducted two systematic reviews to examine the best available scientific evidence on the net effect (i.e., balance of benefits vs. harms) of outdoor and risky active play. Other research and reviews were also consulted. The Position Statement applies to girls and boys (aged 3-12 years) regardless of ethnicity, race, or family socioeconomic status. Children who have a disability or a medical condition should also enjoy active outdoor play in compliance with guidance from a health professional.

CONTEXT

In an era of schoolyard ball bans and debates about safe tobogganing, have we as a society lost the appropriate balance between keeping children healthy and active and protecting them from serious harm? If we make too many rules about what they can and can't do, will we hinder their natural ability to develop and learn? If we make injury prevention the ultimate goal of outdoor play spaces, will they be any fun? Are children safer sitting on the couch instead of playing actively outside? **We need to recognize the difference between danger and risk. And we need to value long-term health and fun as much as we value safety.**

Risk is often seen as a bad word—by parents, neighbours, care providers, insurance providers, schools and municipalities. But in play, risk doesn't mean courting danger—like skating on a half-frozen lake or sending a preschooler to the park alone. It means the types of play children see as thrilling and exciting, where the possibility of physical injury may exist, but they can recognize and evaluate challenges according to their own ability.[1,2] It means giving children the freedom to decide how high to climb, to explore the woods, get dirty, play hide 'n seek, wander in their neighbourhoods, balance, tumble and rough-house, especially outdoors, so they can be active, build confidence, autonomy and resilience, develop skills, solve problems and learn their own limits. It's letting kids be kids—healthier, more active kids.

EVIDENCE

» When children are outside they move more, sit less and play longer[3-12]—behaviours associated with improved cholesterol levels, blood pressure, body composition, bone density, cardiorespiratory and musculoskeletal fitness and aspects of mental, social and environmental health.[13-22]

» Outdoor play is safer than you think!

- The odds of total stranger abduction are about 1 in 14 million based on RCMP reports.[23] Being with friends outdoors may further reduce this number.
- Broken bones and head injuries unfortunately do happen, but major trauma is uncommon. Most injuries associated with outdoor play are minor.[24-31]
- Canadian children are eight times more likely to die as a passenger in a motor vehicle than from being hit by a vehicle when outside on foot or on a bike.[32-34]

» There are consequences to keeping kids indoors—is it really safer?

- When children spend more time in front of screens they are more likely to be exposed to cyber-predators and violence, and eat unhealthy snacks.[35-39]

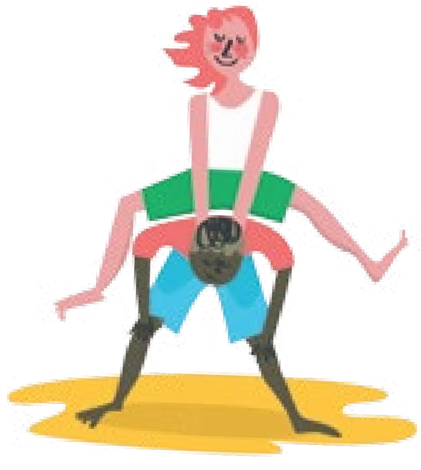

- Air quality indoors is often worse than outdoors, increasing exposure to common allergens (e.g., dust, mould, pet dander), infectious diseases, and potentially leading to chronic conditions.[40-43]
- In the long-term, sedentary behaviour and inactivity elevate odds of developing chronic diseases, including heart disease, type-2 diabetes, some forms of cancer and mental health problems.[44-53]

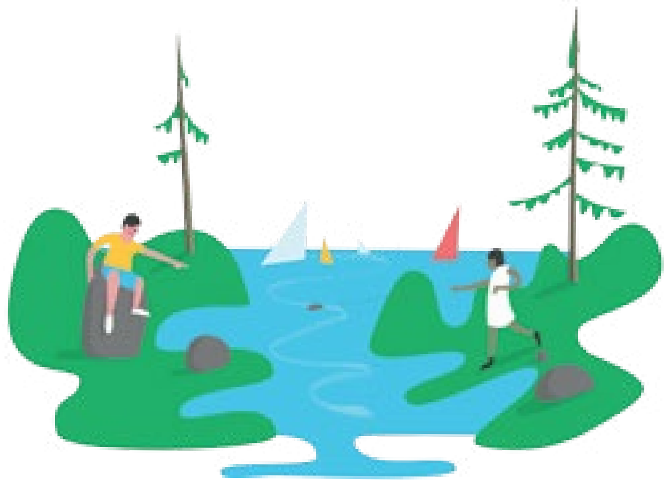

- **Hyper-parenting limits physical activity and can harm mental health.**[54-57]
- **When children are closely supervised outside, they are less active.**[4,58-68]
- **Children are more curious about, and interested in, natural spaces than pre-fabricated play structures.**[69-79] **Children who engage in active outdoor play in natural environments demonstrate resilience, self-regulation and develop skills for dealing with stress later in life.**[80-98]
- **Outdoor play that occurs in minimally structured, free and accessible environments facilitates socialization with peers, the community and the environment, reduces feelings of isolation, builds inter-personal skills and facilitates healthy development.**[4,59,70,76,83,99-103]

RECOMMENDATIONS

- **Parents:** Encourage your children to engage more fully with their outdoor environments in a variety of weather conditions. When children are supported to take risks, they have more fun and learn how to assess and manage risk in all areas of their lives.[2,82,104]
- **Educators and Caregivers:** Regularly embrace the outdoors for learning, socialization and physical activity opportunities, in various weather conditions—including rain and snow. Risky active play is an important part of childhood and should not be eliminated from the school yard or childcare centre.
- **Health Professionals:** Be influential! Promote every child's connection with nature and the outdoors—identify outdoor play resources and partner with municipalities, parks, nature-related organizations, parent groups and children to support this process.
- **Injury Prevention Professionals:** Find a balanced approach to health promotion and protection that considers the long-term dangers of a sedentary lifestyle along with the acute potential for injury.
- **School and Child Care Administrators:** Choose natural elements over pre-fabricated playgrounds and paved areas—and encourage children to play in, and help design, these environments.
- **Media:** Provide balanced reporting—sensationalizing stories about predators and danger elevates fear; cover success stories related to outdoor and risky active play.
- **Attorneys General:** Establish reasonable liability limits for municipal governments—this means Joint and Several Liability Reform.
- **Provincial and Municipal Governments:** Work together to create an environment where Public Entities are protected from frivolous lawsuits over minor injuries related to normal and healthy outdoor risky active play. This protection would no longer restrict Public Entities to using the Canadian Standards Association CAN/CSA Z614 "Children's Playspaces and Equipment" as a guide for the design of outdoor play spaces and as a requirement for the funding of these spaces. An increased investment in natural play spaces in all neighbourhoods is encouraged.
- **Schools and Municipalities:** Examine existing policies and by-laws and reconsider those that pose a barrier to active outdoor play.
- **Federal and Provincial/ Territorial Governments:** Collaborate across sectors to find ways to improve children's access to risky active play in nature and the outdoors.
- **Society:** Recognize that children are competent and capable. Respect parents' assessments of their children's abilities and their decisions to encourage self-directed play in nature and the outdoors. Allow all children to play with and form a lasting relationship with nature on their own terms.

This Position Statement was informed by the best available evidence, interpreted by a group of Canadian experts representing 14 organizations, and reviewed and edited by more than 1,600 stakeholders. Details of the process are published in the *International Journal of Environmental Research and Public Health* [www.mdpi.com/journal/ijerph].

ACKNOWLEDGMENTS

Funding for the development of the Position Statement was provided by:

The Position Statement was developed and is supported by Professor Susan Herrington, MLA, University of British Columbia; Dr. William Pickett, Queen's University, and:

REFERENCES

1. **Ball et al.** Managing risk in play provision: Implementation guide. Play England: London, 2012. p 120.
2. **Sandseter et al.** Children's risky play from an evolutionary perspective: The anti-phobic effects of thrilling experiences. *Evol Psychol* 2011;9:257-84.
3. **Gray et al.** What is the relationship between outdoor time and physical activity, sedentary behaviour, and physical fitness in children? A systematic review. *Int J Environ Res Public Health.* In press.
4. **Brussoni et al.** What is the relationship between risky outdoor play and health in children? A systematic review. *Int J Environ Res Public Health.* In press.
5. **Cooper et al.** Patterns of GPS measured time outdoors after school and objective physical activity in English children: the PEACH project. *Int J Behav Nutr Phys Act* 2010;7:31.
6. **Dunton et al.** Physical and social contextual influences on children's leisure-time physical activity: an ecological momentary assessment study. *J Phys Act Health* 2011;8(Suppl. 1): 2011.
7. **Klinker et al.** Context-specific outdoor time and physical activity among school-children across gender and age: using accelerometers and GPS to advance methods. *Front Public Health* 2014;2:20.
8. **Raustorp et al.** Accelerometer measured level of physical activity indoors and outdoors during preschool time in Sweden and the United States. *J Phys Act Health* 2012;9:801-808.
9. **Smith et al.** Assessment of physical activity levels of 3rd and 4th grade children using pedometers during physical education class. *J Res* 2009;4:73-9.
10. **Skala et al.** Environmental characteristics and student physical activity in PE class: findings from two large urban areas of Texas. *J Phys Act Health* 2012;9:481-91.
11. **Vanderloo et al.** Physical activity among preschoolers during indoor and outdoor childcare play periods. *Appl Physiol Nutr Metab* 2013;38:1173-5.
12. **Wheeler et al.** Greenspace and children's physical activity: a GPS/GIS analysis of the PEACH project. *Prev Med* 2010;51:148-52.
13. **Andersen et al.** Physical activity and clustered cardiovascular risk in children: a cross-sectional study (the European Youth Heart Study). *Lancet* 2006;368:299-304.
14. **Ekelund et al.** Moderate to vigorous physical activity and sedentary time and cardiometabolic risk factors in children and adolescents. *JAMA* 2012;307(7):704-12.
15. **Janssen I, LeBlanc A.** Systematic review of the health benefits of physical activity and fitness in school-aged children and youth. *Int J Behav Nutr Phys Act* 2010;7:40.
16. **Strong et al.** Evidence-based physical activity for school-aged youth. *J Pediatr* 2005;146:732-7.
17. **Duncan et al.** The effect of green exercise on blood pressure, heart rate and mood state in primary school children. *Int J Environ Res Public Health* 2014;11:3678-88.
18. **Kemper et al.** A fifteen-year longitudinal study in young adults on the relation of physical activity and fitness with the development of the bone mass: the Amsterdam Growth and Health Longitudinal Study. *Bone* 2000;27:847-53.
19. **Hind K, Burrows M.** Weight-bearing exercise and bone mineral accrual in children and adolescents: a review of controlled trials. *Bone* 2007;40:14-27.
20. **Tremblay et al.** Systematic review of sedentary behaviour and health indicators in school-aged children and youth. *Int J Behav Nutr Phys Act* 2011;8:98.
21. **Larouche R.** The environmental and population health benefits of active transport: A review. In G. Liu (Ed.) Greenhouse Gases – Emissions, Measurement and Management. InTech: Rijeka, Croatia, 2012. p 413-40.
22. **Friedman et al.** Impact of changes in transportation and commuting behaviors during the 1996 Summer Olympic Games in Atlanta on air quality and childhood asthma. *JAMA* 2001;285:897-905.
23. **Dalley ML, Ruscoe J.** The abduction of children by strangers in Canada: Nature and scope. Royal Canadian Mounted Police. 2003. http://www.rcmp-grc.gc.ca/pubs/omc-ned/abd-rapt-eng.htm
24. **Fuselli P, Yanchar NL.** Preventing playground injuries. *Paediatr Child Health* 2012;17:328.
25. **Canadian Institute for Health Information.** National Trauma Registry Minimum Data Set, 1994-1995 to 2012-2013. http://tinyurl.com/pv3p2k4
26. **Rubie-Davies CM, Townsend MAR.** Fractures in New Zealand elementary school settings. *J Sch Health* 2007;77:36-40.
27. **Nauta et al.** Injury risk during different physical activity behaviours in children: A systematic review with bias assessment. *Sport Med* 2015;45;327-36.
28. **Public Health Agency of Canada Child and youth injury in review, 2009 edition: Spotlight on consumer product safety.** Public Health Agency of Canada: Ottawa, 2009.
29. **Belechri et al.** Sports injuries among children in six European Union countries. *Eur J Epidemiol* 2001;17:1005-12.
30. **Sahai et al.** Quantifying the iceberg effect for injury: using comprehensive community health data. *Can J Public Health* 2005;96:328-32.
31. **Howard et al.** School playground surfacing and arm fractures in children: a cluster randomized trial comparing sand to wood chip surfaces. *PLoS Med* 2009;6(12):e1000195.
32. **Public Health Agency of Canada.** Injury in Review, 2012 Edition: Spotlight on Road and Transport Safety. Public Health Agency of Canada: Ottawa, 2012.
33. **Rothman et al.** Motor vehicle-pedestrian collisions and walking to school: the role of the built environment. *Pediatrics* 2014;133:776-84.
34. **DiMaggio C, Li G.** Effectiveness of a safe routes to school program in preventing school-aged pedestrian injury. *Pediatrics* 2013;131:290-6.
35. **Dombrowski et al.** Protecting children from online sexual predators: technological, psychoeducational, and legal considerations. *Prof Psychol Res Proc* 2004;35:65-73.
36. **Mazowita B, Vézina M.** Police-reported cybercrime in Canada 2012. Juristat Catalogue no. 85-002-X. Statistics Canada, 2014.
37. **Litwiller BJ, Brausch AM.** Cyber bullying and physical bullying in adolescent suicide: the role of violent behavior and substance use. *J Youth Adolesc* 2013;42:675-84.
38. **Browne KD, Hamilton-Giachritsis C.** The influence of violent media on children and adolescents: a public-health approach. *Lancet* 2005;365:702-10.
39. **Borghese et al.** Independent and combined associations of total sedentary time and television viewing time with food intake patterns of 9- to 11-year-old Canadian children. *Appl Physiol Nutr Metab* 2014;39:937-43.
40. **Spengler JD, Sexton K.** Indoor air pollution: a public health perspective. *Science* 1983;221:9-17.
41. **Jones, AP.** Asthma and domestic air quality. *SocSci Med* 1991;47:755-64.
42. **DellaValle et al.** Effects of ambient pollen concentrations on frequency and severity of asthma symptoms among asthmatic children. *Epidemiol* 2012;23:55-63.
43. **World Health Organization.** Burden of disease from household air pollution for 2012. Available at http://www.who.int/phe/health_topics/outdoorair/databases/HAP_BoD_results_March2014.pdf [Cited Mar 24, 2015].
44. **Lee et al.** Effect of physical activity on major non-communicable diseases worldwide: an analysis of burden of disease and life expectancy. *Lancet* 2012;380:219-29.
45. **Pahkala et al.** Association of physical activity with vascular endothelial function and intima-media thickness. *Circulation* 2011;124:1956-63.
46. **Raitakari et al.** Effects of persistent physical activity and inactivity on coronary risk factors in children and young adults: the Cardiovascular Risk in Young Finns Study. *Am J Epidemiol* 1994;140:195-205.
47. **Tuomiletho et al.** Prevention of type 2 diabetes mellitus by changes in lifestyle among subjects with impaired glucose tolerance. *N Engl J Med* 2001;344:1343-50.
48. **Knowler et al.** Reduction in the incidence of type 2 diabetes with lifestyle intervention or metformin. *N Engl J Med* 2002;346:393-403.
49. **Mammen G, Faulkner G.** Physical activity and the prevention of depression: a systematic review of prospective studies. *Am J Prev Med* 2013;45:649-57.
50. **Paffenbarger et al.** Physical activity, all-cause mortality, and longevity of college alumni. *N Engl J Med* 1986;314:605-23.
51. **Paffenbarger et al.** The association of changes in physical activity

level and other lifestyle characteristics with mortality among men. *N Engl J Med* 1993;328:538-45.

52. **U.S. Department of Health and Human Services.** Physical Activity Guidelines Advisory Committee Report, 2008. Washington, DC: U.S. Department of Health and Human Services, 2008.

53. **Biswas et al.** Sedentary time and its association with risk for disease incidence, mortality, and hospitalization in adults: a systematic review and meta-analysis. *Ann Intern Med* 2015;162:123-32.

54. **Janssen I.** Hyper-parenting is negatively associated with physical activity among 7-12 year olds. *Prev Med* 2015;73:55-9.

55. **Ginsburg KR.** The importance of play in promoting healthy child development and maintaining strong parent-child bonds. *Pediatrics* 2007;119:182-91.

56. **Schiffrin et al.** Helping or hovering? The effects of helicopter parenting on college students' well-being. *J Child Fam Stud* 2014;23:548-57.

57. **LeMoyne T, Buchanan T.** Does "hovering" matter? Helicopter parenting and its effect on well-being. *Sociological Spectrum* 2011;31:399-418.

58. **Gester S.** Urban children's access to their neighborhoods: Changes over three generations. *Environ Behav* 1991;23:70-85.

59. **Hillman et al.** One false move: A study of children's independent mobility. London: PSI Publishing; 1990.

60. **O'Brien et al.** Children's independent spatial mobility in the urban public realm. *Childhood* 2001;7:257-77.

61. **Shaw et al.** Children's independent mobility: a comparative study in England and Germany (1971-2010). London: Policy Studies Institute; 2013.

62. **Kirby et al.** Parental and peer influences on physical activity among Scottish adolescents: a longitudinal study. *J Phys Act Health* 2011;8:785-93.

63. **Page et al.** Independent mobility in relation to weekday and weekend physical activity in children aged 10–11 years: The PEACH Project. *Int J Behav Nutr Phys Act* 2009;6:2.

64. **Page et al.** Independent mobility, perceptions of the built environment and children's participation in play, active travel and structured exercise and sport: the PEACH Project. *Int J Behav Nutr Phys Act* 2010;7:17.

65. **Stone et al.** The freedom to explore: examining the influence of independent mobility on weekday, weekend and after-school physical activity behaviour in children living in urban and inner-suburban neighbourhoods of varying socioeconomic status. *Int J Behav Nutr Phys Act* 2014;11:5.

66. **Schoeppe et al.** Associations between children's independent mobility and physical activity. *BMC Public Health* 2014;14:91.

67. **Mitra et al.** Do parental perceptions of the neighbourhood environment influence children's independent mobility? Evidence from Toronto, Canada. *Urban Studies* 2014;51(16):3401-19.

68. **Floyd et al.** Park-based physical activity among children and adolescents. *Am J Prev Med* 2011;41:258–65.

69. **Jones O.** True geography quickly forgotten, giving away to an adult-imagined universe. Approaching the otherness of childhood. *Child Geogr* 2008;6:195-212.

70. **Aasen et al.** The outdoor environment as a site for children's participation, meaning-making and democratic learning: examples from Norwegian kindergartens. *Education* 2009;37:5-13.

71. **Dyment J, O'Connell TS.** The impact of playground design on play choices and behaviors of pre-school children. *Child Geogr* 2013;11:263-80.

72. **Mahidin AMM, Maulan S.** Understanding children preferences of natural environment as a start for environmental sustainability. *Procedia – Soc Behav Sci* 2012;38:324-33.

73. **Fjortoft I, Sageie J.** The natural environment as a playground for children: Landscape description and analyses of a natural playscape. *Landscape Urban Plan* 2000;48;83-97.

74. **Luchs A, Fikus M.** A comparative study of active play on differently designed playgrounds. *J Adventure Educ Outdoor Learn* 2013;13:206–22.

75. **Cloward Drown KK, Christensen KM.** Dramatic play affordances of natural and manufactured outdoor settings for preschool-aged children. *Children Youth Environ* 2014;24:53-77.

76. **Dowdell et al.** Nature and its influence on children's outdoor play. *Aust J Outdoor Ed* 2011;15(2):12.

77. **Reed et al.** A repeated measures experiment of green exercise to improve self-esteem in UK school children. *PLoS ONE*. 2013;8:7.

78. **Herrington S, Studtmann K.** Landscape interventions: New directions for the design of children's outdoor play environments. *Landscape Urban Plan* 1998;42:191-205.

79. **Herrington S.** The received view of play and the subculture of infants. *Landsc J* 1997;16:149–60.

80. **Veitch et al.** Where do children usually play? A qualitative study of parents' perceptions of influences on children's active free-play. *Health Place* 2006;12:383-93.

81. **Brussoni et al.** Risky play and children's safety: Balancing priorities for optimal child development. *Int J Environ Res Public Health* 2012;9:3134-8.

82. **Hüttenmoser M.** Children and their living surroundings: Empirical investigation into the significance of living surroundings for the everyday life and development of children. *Child Environ* 1995;12:403-13.

83. **Prezza et al.** The influence of psychosocial and environmental factors on children's independent mobility and relationship to peer frequentation. *J Community Appl Soc Psychol* 2001;11:435-50.

84. **Becker et al.** Physical activity, self-regulation, and early academic achievement in preschool children. *Early Educ Dev* 2014;25:56-70.

85. **Kahn P, Kellert S.** Children and Nature: Psychological, Socio-cultural, and Evolutionary Investigations. Boston, MA: MIT Press, 2002.

86. **Bingley A, Milligan C.** Climbing trees and building dens: Mental health and well-being in young adults and the long-term experience of childhood play experience. London, U.K.: Lancaster University, Institute for Health Research, 2004. Available at http://escalate.ac.uk/downloads/4725.pdf.

87. **Greffrath et al.** Centre-based and expedition-based (wilderness) adventure experiential learning personal effectiveness: an explorative enquiry. *Leisure Studies* 2011;30:345-64.

88. **Korpela et al.** Restorative experience, self-regulation, and children's special place preferences. *J Environ Psychol* 2002;22:387-98.

89. **Sandseter.** Risky play and risk management in Norwegian preschools - A qualitative observational study. *Saf Sci Monit* 2009;13:1–12.

90. **Mikkelsen MR, Christensen P.** Is children's independent mobility really independent? A study of children's mobility combining ethnography and GPS/mobile phone technologies. *Mobilities* 2009;4:37–58.

91. **Lavrysen et al.** Risky-play at school. Facilitating risk perception and competence in young children. *Eur. Early Child. Educ* 2015; (in press).

92. **Ungar M.** Too safe for their own good. Toronto: McClelland & Stewart; 2007.

93. **Gray P.** The decline of play and the rise of psychopathology in children and adolescents. *Am J Play* 2011;3:443-63.

94. **Twenge et al.** Birth cohort increases in psychopathology among young Americans, 1938-2007: A cross-temporal meta-analysis of the MMPI. *Clin Psychol Rev* 2010;30:145-54.

95. **Twenge JM.** The age of anxiety? Birth cohort change in anxiety and neuroticism, 1952-1993. *J Pers Soc Psychol* 2000;79:1007-21.

96. **Kochanowski L, Carr V.** Nature playscapes as contexts for fostering self-determination. *Child Youth Environ* 2014;24(2):146-67.

97. **McArdle et al.** Does a nurturing approach that uses an outdoor play environment build resilience in children from a challenging background? J *Adventure Ed Outdoor Learn* 2013;13(3):238-54.

98. **Canning N.** 'Where's the bear? Over there!' – creative thinking and imagination in den making. *Early Child Dev Care* 2013;183:1042-53.

99. **Malone K, Rudner J.** Global perspectives on children's independent mobility: a socio-cultural comparison and theoretical discussion of children's lives in four countries in Asia and Africa. *Global Studies Childhood* 2011;1:243-59.

100. **Joshi et al.** Children's journey to school: spatial skills, knowledge and perceptions of the environment. *Br J Dev Psychol* 1999;19:125-39.

101. **Rissotto A, Tonucci F.** Freedom of movement and environmental knowledge in elementary school children. *J Environ Psychol* 2002;22:65-77.

102. **Bixler et al.** Environmental socialization: quantitative tests of the childhood play hypothesis. *Environ Behav* 2002;34:795-818.

103. **Pacilli et al.** Children and the public realm: antecedents and consequences of independent mobility in a group of 11 – 13-year-old Italian children. *Child Geogr* 2013l;11:377-93.

104. **Gill T.** No fear: growing up in a risk averse society. London: Calouste Gulbenkian Foundation, 2007.
